# Long-term culture of patient-derived cardiac organoids recapitulated Duchenne muscular dystrophy cardiomyopathy and disease progression

**DOI:** 10.3389/fcell.2022.878311

**Published:** 2022-08-11

**Authors:** Vittoria Marini, Fabiola Marino, Flaminia Aliberti, Nefele Giarratana, Enrico Pozzo, Robin Duelen, Álvaro Cortés Calabuig, Rita La Rovere, Tim Vervliet, Daniele Torella, Geert Bultynck, Maurilio Sampaolesi, Yoke Chin Chai

**Affiliations:** ^1^ Translational Cardiomyology Laboratory, Stem Cell Biology and Embryology, Department of Development and Regeneration, KU Leuven, Leuven, Belgium; ^2^ Department of Experimental and Clinical Medicine, Magna Graecia University, Catanzaro, Italy; ^3^ Fondazione IRCCS Policlinico San Matteo, Center for Inherited Cardiovascular Diseases, Transplant Research Area, Human Anatomy Unit, Department of Public Health, Experimental and Forensic Medicine, University of Pavia, Pavia, Italy; ^4^ Genomics Core Leuven, KU Leuven, Leuven, Belgium; ^5^ Laboratory of Molecular and Cellular Signaling, Department of Cellular and Molecular Medicine and Leuven Kanker Institute, KU Leuven, Leuven, Belgium; ^6^ Histology and Medical Embryology Unit, Department of Anatomy, Histology, Forensic Medicine and Orthopedics, Sapienza University of Rome, Rome, Italy

**Keywords:** Duchenne muscular dystrophy, induced pluripotent stem cells, cardiomyopathy, cardiac organoids, disease modeling, aberrant adipogenesis, fibrosis

## Abstract

Duchenne Muscular Dystrophy (DMD) is an X-linked neuromuscular disease which to date is incurable. The major cause of death is dilated cardiomyopathy however, its pathogenesis is unclear as existing cellular and animal models do not fully recapitulate the human disease phenotypes. In this study, we generated cardiac organoids from patient-derived induced pluripotent stem cells (DMD-COs) and isogenic-corrected controls (DMD-Iso-COs) and studied if DMD-related cardiomyopathy and disease progression occur in the organoids upon long-term culture (up to 93 days). Histological analysis showed that DMD-COs lack initial proliferative capacity, displayed a progressive loss of sarcoglycan localization and high stress in endoplasmic reticulum. Additionally, cardiomyocyte deterioration, fibrosis and aberrant adipogenesis were observed in DMD-COs over time. RNA sequencing analysis confirmed a distinct transcriptomic profile in DMD-COs which was associated with functional enrichment in hypertrophy/dilated cardiomyopathy, arrhythmia, adipogenesis and fibrosis pathways. Moreover, five miRNAs were identified to be crucial in this dysregulated gene network. In conclusion, we generated patient-derived cardiac organoid model that displayed DMD-related cardiomyopathy and disease progression phenotypes in long-term culture. We envision the feasibility to develop a more complex, realistic and reliable *in vitro* 3D human cardiac-mimics to study DMD-related cardiomyopathies.

## Introduction

Duchenne Muscular Dystrophy (DMD) is one of the most common muscular dystrophies which affects 1:5000 live male births (Yiu and Kornberg, 2015). It is a progressive X-linked genetic disorder caused by mutations within the *DMD* gene, which results in a complete absence of Dystrophin (DYS) protein expression (Muntoni et al., 2003; Flanigan, 2014; Loboda and Dulak, 2020). The absence of DYS leads to muscle weakness and wasting, owing to the loss of muscle membrane integrity and susceptibility to stress-induced damages (Lin et al., 2015). In recent years, the use of respiratory assist devices and non-invasive positive pressure ventilation have increased the life expectancy of DMD patients, nevertheless this has contributed to the rise of previously unknown late-stages DMD complications, such as dilated cardiomyopathy (DCM) (Kamdar and Garry, 2016; Breuls et al., 2021).

DMD-associated DCM is characterized by initial cardiomyocyte degeneration attributed to the inflammatory response, which leads to the replacement of heart muscle with fat and connective tissue (i.e. fibrosis of the left-ventricular (LV) myocardial wall) and thus the reduction of cardiac wall thickness (Finsterer and Stollberger, 2003; Law et al., 2020). Due to the latter, the myocardium becomes more sensitive to pressure overload causing LV dilatation, cardiac contractility reduction and eventually congestive heart failure (Luk et al., 2009; Fayssoil et al., 2010; McNally and Mestroni, 2017). Although DCM represents the major cause of mortality in DMD patients, no great research attention has been directed to DCM–partly due to limited accessibility to human cardiac tissues and the intrinsic limitation of two-dimensional (2D) cardiomyocyte culture in recapitulating human three-dimensional (3D) pathophysiology (Lin et al., 2015; Quattrocelli et al., 2015; Law et al., 2020). Similarly, DMD animal models (*mdx* mice and canine DMD models) do not fully resemble human DMD features and its disease progression, mainly due to inter-species variations. It is therefore imperative to develop 3D human cardiac-mimics of DMD-relevance to bridge this scientific gap (McGreevy et al., 2015; Filippo Buono et al., 2020; Jensen and Teng, 2020; Zhao et al., 2021).

Organoids are *in vitro* self-organized 3D cellular structures derived from either primary tissues or stem cells [e.g. embryonic (ESCs) or induced pluripotent stem cells (iPSCs), and primary stem cells] differentiated into designated functional cell types. They possess organotypic structures including the cytoarchitecture and the mechanisms involved in the cell behavior and fate within the specific tissue (Velasco et al., 2020; Heydari et al., 2021; Scalise et al., 2021). The advent of iPSC and CRISPR/Cas9 technologies represent a paramount breakthrough for patient-specific model generation, enabling the development of iPSC-derived cardiomyocyte (CM)-based 3D models and the isogenic controls, which are widely used to study patient-specific cardiac diseases *in vitro* (Filippo Buono et al., 2020; Richards et al., 2020)*.* Although 3D cardiac models were used for investigating abnormal mechanical and electromechanical properties of DMD CMs (Caluori et al., 2019; Jelinkova et al., 2020), as to our knowledge, the organoid technology with proper isogenic control has not been used to model DMD cardiomyopathies*.* Given that, this study focused on the development of 3D cardiac organoids (COs) from DMD patient-derived iPSC (DMD-COs) and its mutation-corrected isogenic iPSC controls (DMD-Iso-COs), and studied if these human cardiac-mimics could reproduce DMD-related cardiomyopathy and disease progression in 3D *via* long-term culture.

## Materials and methods

### Cell cultures

Duchenne Muscular Dystrophy human iPSC (DMD-hiPSC) was obtained from DMD patient’s fibroblasts carrying a point mutation in exon 35 (c.4 996C>T; p.Arg1,666X) of the *DMD* gene that leads to a premature stop codon (Duelen et al., 2022). The DMD isogenic control (DMD-Iso-hiPSC) was generated through CRISPR/Cas9 gene editing from the *S. pyogenes* system (5’-NGG PAM) as previously described (Ran et al., 2013; Duelen et al., 2022). The healthy control iPSC (HC-hiPSC) line was a gift from Prof. P. Jennings (Medizinische Universität Innsbruck, Austria) to Stem Cell Institute Leuven, KU Leuven and generated by SeV-based reprogramming of male donor fibroblasts (SBAD2). Human iPSC lines were cultured feeder-free on Geltrex LDEV-Free hESC-Qualified Reduced Growth Factor Basement Membrane Matrix and maintained in Essential 8 Flex Basal Medium (Thermo Fisher Scientific) supplemented with Essential 8 Flex Supplement (50x, Thermo Fisher Scientific) and penicillin–streptomycin (0.1%, Thermo Fisher Scientific), at 37°C under normoxic conditions (21% O_2_ and 5% CO_2_). Colonies were routinely passaged non-enzymatically with 0.5 mM EDTA in Phosphate-Buffered Saline (PBS, Thermo Fisher Scientific). The use of human samples from DMD subjects for experimental purposes and protocols in the present study was approved by the Ethics Committee of the University Hospitals Leuven (S55438 and S65190, respectively).

### Monolayer-based cardiac differentiation of human iPSCs

DMD-, DMD-Iso- and HC-hiPSC lines were differentiated into functional cardiomyocytes (CMs) according to a monolayer-based cardiac differentiation protocol, as previously described (Burridge et al., 2014). Briefly, prior to differentiation, the DMD-, DMD-Iso- and HC-hiPSC lines were suspended into small colonies and subsequently cultured on Matrigel Growth Factor Reduced (GFR) Basement Membrane Matrix layer (Corning) in complete Essential 8 Flex Medium at 37°C under hypoxic conditions (5% O_2_ and 5% CO_2_) for 3 days, in order to obtain the pre-optimized targeted confluency of 85%. Mesoderm differentiation (day 0) was induced using 6 µM CHIR99021 (Axon Medchem) for 48 h in a chemically defined medium consisting of RPMI 1640 (Thermo Fisher Scientific), 500 μg/ml rice-derived recombinant human albumin and 213 μg/ml L-ascorbic acid 2-phosphate (Sigma-Aldrich). After 24 h of CHIR99021 stimulation, the cells were transferred from hypoxia to normoxia. On day 2 of differentiation, iPSC-derived mesodermal cells were fed with basal medium supplemented with 4 µM IWR-1 (Sigma-Aldrich) for 48 h, to induce cardiac progenitor cell differentiation. From day 4 onwards, medium was refreshed every 2 days with CM Maintenance Medium (RPMI 1640, rice-derived recombinant human albumin and L-ascorbic acid 2-phosphate). Contracting CMs appeared at day 8 or 9 of cardiac differentiation.

### Agarose microwell culture insert fabrication

A 3% agarose (Invitrogen) gel solution was prepared in PBS. The powder was fully dissolved by heating in microwave oven and the agarose microwells were fabricated in sterile conditions. In brief, the heated agarose solution was added into a custom-made 3D printed micropillar molds (in 24-well plate format). Upon cooling at room temperature for 10 min, the agarose was removed from the molds, thus creating 24 culture inserts each consisting of 137 microwells (diameter × height = 500 × 700 μm). The culture inserts were transferred into a 24-well plate and equilibrated in PBS overnight at 37°C under normoxia conditions (5% O_2_ and 5% CO_2_).

### Generation of cardiac organoids

After reaching confluency, the DMD-, DMD-Iso- and HC-hiPSC lines were detached using 0.5 mM EDTA at 37°C and resuspended in Essential 8TM medium supplemented with Revitacel^™^ Supplement (dilution 1:100, Thermo Fisher Scientific). After cell count, the hiPSCs were resuspended in 1 ml of Essential 8 Flex Basal Medium (Thermo Fisher Scientific) and were plated in agarose inserts at two different cell densities, 5 × 10^3^ cells/microwell and 1 × 10^4^ cells/microwell respectively. The plates were centrifuged for 10 min at 1,200 rpm to facilitate sedimentation of cells in the microwells. Subsequently, 1 ml of fresh Essential 8 Flex Basal Medium was added to completely cover the microwell area and incubated at 37°C under hypoxic conditions (5% O_2_ and 5% CO_2_) to promote embryoid bodies (EBs) formation. The medium was refreshed every day for 3 days and cardiac differentiation of the EBs into cardiac organoids (COs) was initiated as described above for the monolayer cardiomyocyte differentiation protocol. On day 5, the COs were transferred from the agarose molds to an ultra-low attachment 6-well plate (Costar, Corning) and dynamic culture was carried out using an orbital shaker at 75 rpm in CM maintenance medium until day 93. The media was changed every 2 days. Contracting COs start to appear from day 8 of the differentiation protocol. The samples were collected on day 10, 14, 28, 56 and 93 for subsequent analyses.

### Hematoxylin and Eosin (H&E), Picro-Sirius Red (PSR), and BODIPY stainings

At different time points, the COs were fixed with 4% paraformaldehyde (PFA; Polysciences) for 30 min at room temperature and subsequently embedded in cryogel (Tissue- Tek ^®^ O.C.T. ^™^ Compound). The samples were snap-frozen in liquid nitrogen and stored at −80°C until cryosectioning. The samples were sectioned at the thickness of 6 µm using the HM525 NX Cryostat (Thermo Scientific) and stored at −20°C prior to analysis. For H&E staining, the cryosections were stained in Harris hematoxylin solution (Sigma-Aldrich), counterstained in eosin solution (0.1% erithrosin extra bluish Sigma-Aldrich in 70% ethanol) and mounted with DPX mountant (Sigma) upon dehydration according to routine protocols. For PSR staining, the cryosections were stained for collagen content using the Vitro View^™^ Picro-Sirius Red Stain Kit (Cat. No. VB-3017) according to the manufacturer’s instructions (Giarratana et al., 2020). The nuclei were counterstained with Weigert’s Hematoxylin Solution and mounted with DPX mountant (Sigma-Aldrich). Lipid droplets deposition was detected by BODIPY staining. In brief, the BODIPY^™^ 493/503 4,4-Difluoro-1,3,5,7,8-Pentamethyl-4-Bora-3a,4a-Diaza-s-Indacene (Invitrogen) powder was dissolved in DMSO at the concentration of 1.3 mg/ml. The cryosections were incubated with the BODIPY solution, diluted 1:2500 in PBS, for 15 min at room temperature and subsequently mounted with Antifade Mounting Medium with DAPI (VECTASHIELD^®^). All images were acquired using Axiocam MRm microscope (Zeiss).

### RNA isolation and quantitative real-time PCR

For quantitative Reverse Transcription Polymerase Chain Reaction (RT-qPCR) assays, total RNA was isolated through Purelink^®^ RNA mini kit (Thermo Fisher Scientific) and treated with TurboTM DNA-free kit (Thermo Fisher Scientific) to purify RNA samples. 1 μg RNA was reverse-transcribed using Superscript III Reverse Transcriptase First-Strand Synthesis SuperMix (Thermo Fisher Scientific). Thermal cycler setting: 25°C 10 min, 50°C 30 min, 85°C 5 min, 37°C 20 min incubation with *E. Coli* RNAse H. A 384-well plate was prepared using Platinum SYBR Green QPCR SuperMix-UDG (Thermo Fisher Scientific) as SYBR Green on 1:5 diluted cDNA. The RT-qPCR was performed by Viia7 384-plate reader (Thermo Fisher Scientific; final primer concentration, 100 nM; final volume, 10 μl; thermal profile, 95°C 15 s, 60°C 60 s, 40×). The oligonucleotide primer sequences are listed in Table 1. Delta Ct (ΔCt) values were calculated by subtracting the Ct values from the genes of interest with the Ct values of the housekeeping genes (GAPDH).

**TABLE 1:** List of primer used for gene expression analysis.

Gene	Primer sequence	Gene	Primer sequence
*TNNI1*	FW: 5′-CCC​AGC​TCC​ACG​AGG​ACT​GAA​CA-3′	*MYL3*	FW: 5′-TCA​CAC​CTG​AGC​AGA​TTG​AAG​A-3′
	RV: 5′-TTT​GCG​GGA​GGC​AGT​GAT​CTT​GG-3′		RV: 5′-GCT​GGA​GCA​TAG​GCA​GGA​AAG-3′
*TNNC1*	FW: 5′-TGC​TGC​AGG​CTA​CAG​GCG​AG-3′	*TPM1*	FW: 5′-TTG​AGA​GTC​GAG​CCC​AAA​AAG-3′
	RV: 5′-TCG​ATG​CGG​CCG​TCG​TTG​TT-3′		RV: 5′-CAT​ATT​TGC​GGT​CGG​CAT​CTT-3′
*TNNI3*	FW: 5′-GGA​ACC​TCG​CCC​TGC​ACC​AG-3′	*MYOM1*	FW: 5′-GAG​TCG​ATA​TGG​GAT​GCA​CAC-3′
	RV: 5′-GCG​CGG​TAG​TTG​GAG​GAG​CG-3′		RV: 5′-TCC​TTT​AAC​ATT​CAT​CGC​CGA​G-3′
*ACTN2*	FW: 5′-CTC​AAA​GCT​TAA​CAA​GGA​TGA​CC-3′	*MYBPC3*	FW: 5′-AGC​GGG​TGG​AGT​TTG​AGT​G-3′
	RV: 5′-GTG​GTA​GAA​GCA​AGA​GAC​GTA-3′		RV: 5′-GCG​ATG​CTC​TGG​TAC​ACC​TC-3′
*MYL2*	FW: 5′-TTG​GGC​GAG​TGA​ACG​TGA​AAA-3′	*TNNC2*	FW: 5′-TGA​TGG​TGC​GCC​AGA​TGA​AAG-3′
	RV: 5′-CCG​AAC​GTA​ATC​AGC​CTT​CAG-3′		RV: 5′-TGC​ATT​CCT​GTC​GAA​GAT​GCG-3′
*MYH7*	FW: 5′-ACT​GCC​GAG​ACC​GAG​TAT​G-3′	*IRX4*	FW: 5′-GGC​TCC​CCA​GTT​CTT​GAT​GG-3′
	RV: 5′-GCG​ATC​CTT​GAG​GTT​GTA​GAG​C-3′		RV: 5′-TAG​ACC​GGG​CAG​TAG​ACC​G-3′
*MYH6*	FW: 5′-GCC​CTT​TGA​CAT​TCG​CAC​TG-3′	*FN1*	FW: 5′-CGG​TGG​CTG​TCA​GTC​AAA​G-3′
	RV: 5′-CGG​GAC​AAA​ATC​TTG​GCT​TTG​A-3′		RV: 5′-AAA​CCT​CGG​CTT​CCT​CCA​TAA-3′
*MYL7*	FW: 5′-ACA​TCA​TCA​CCC​ACG​GAG​AAG​AGA-3′	*COL3A1*	FW: 5′-TTG​AAG​GAG​GAT​GTT​CCC​ATC​T-3′
	RV: 5′-ATT​GGA​ACA​TGG​CCT​CTG​GAT​GGA-3′		RV: ACA​GAC​ACA​TAT​TTG​GCA​TGG​TT-3′
*HCN4*	FW: 5′-GAA​CAG​GAG​AGG​GTC​AAG​TCG-3′	*COL1A2*	FW: 5′-GGC​CCT​CAA​GGT​TTC​CAA​GG-3′
	RV: 5′-CAT​TGA​AGA​CAA​TCC​AGG​GTG​T-3′		RV: 5′-CAC​CCT​GTG​GTC​CAA​CAA​CTC-3′
*ACTN1*	FW: 5′-CCA​CCC​TCT​CGG​AGA​TCA​AG-3′	*GAPDH*	FW: 5′-TCA​AGA​AGG​TGG​TGA​AGC​AGG-3′
	RV: 5′-TCC​CTT​CGC​TTC​TGA​GTT​AGG-3′		RV: 5′-ACC​AGG​AAA​TGA​GCT​TGA​CAA​A-3′
*TNNT2*	FW: 5′-GGA​GGA​GTC​CAA​ACC​AAA​GCC-3′	*DMD (Dp427m)*	FW: 5′-ATG​CTT​TGG​TGG​GAA​GAA​GT-3′
	RV: 5′-TCA​AAG​TCC​ACT​CTC​TCT​CCA​TC-3′		RV: 5′-GGG​CAT​GAA​CTC​TTG​TGG​AT-3′
*OGN*	FW: 5′-TCT​ACA​CTT​CTC​CTG​TTA​CTG​CT-3′	*HEY*	FW: 5′-GCC​CGC​CCT​TGT​CAG​TAT​C-3′
	RV: 5′-GAG​GTA​ATG​GTG​TTA​TTG​CCT​CA-3′		RV: 5′-CCA​GGG​TCG​GTA​AGG​TTT​ATT​G-3′
*MPG*	FW: 5′-TCC​GAG​AAC​GCT​CTA​AGC​CT-3′	*COL1A1*	FW: 5′-GAG​GGC​CAA​GAC​GAA​GAC​ATC-3′
	RV: 5′-GCA​AAG​TCT​GTA​GTC​ATC​ACA​GG-3′		RV: 5′-CAG​ATC​ACG​TCA​TCG​CAC​AAC
		*IGF1*	FW: 5′-GCT​CTT​CAG​TTC​GTG​TGT​GGA-3′
			RV: 5′-GCC​TCC​TTA​GAT​CAC​AGC​TCC-3′
*ACTC1*	FW: 5′-TCC​CAT​CGA​GCA​TGG​TAT​CAT-3′	*LEPR*	FW: 5′-ACC​TCT​GGT​TCC​CCA​AAA​AGG-3′
	RV: 5′-GGT​ACG​GCC​AGA​AGC​ATA​CA-3′		RV: 5′-TTG​GCA​CAG​GCA​CAA​GAC​AT-3′
*DCN*	FW: 5′-ATG​AAG​GCC​ACT​ATC​ATC​CTC​C-3′	*MATN4*	FW: 5′-TGC​GTC​CAC​AAA​ACT​TCG​AG-3′
	RV: 5′-GTC​GCG​GTC​ATC​AGG​AAC​TT-3′		RV: 5′-GGA​GAA​GCT​GTG​CTC​CAC​C-3′
*S100A6*	FW: 5′-GGG​AGG​GTG​ACA​AGC​ACA​C-3′	*LUM*	FW: 5′-GGA​TTG​GTA​AAC​CTG​ACC​TTC​AT-3′
	RV: 5′-AGC​TTC​GAG​CCA​ATG​GTG​AG-3′		RV: 5′-GAT​AAA​CGC​AGA​TAC​TGC​AAT​GC-3′
*SSPN*	FW: 5′-TGT​GTC​TCA​TAT​CAG​GTT​GAC​GA-3′	*MYL9*	FW: 5′-TCT​TCG​CAA​TGT​TTG​ACC​AGT-3′
	RV: 5′-CAA​GAG​TCG​AGT​GTG​GTC​TCA-3′		RV: 5′-GTT​GAA​AGC​CTC​CTT​AAA​CTC​CT-3′
*ELN*	FW: 5′-GCA​GGA​GTT​AAG​CCC​AAG​G-3′	*NEXN*	FW: 5′-AGC​GTG​AAT​TAG​CAA​AAA​GGG​C-3′
	RV: 5′-TGT​AGG​GCA​GTC​CAT​AGC​CA-3′		RV: 5′-CCT​TGA​GAG​ATG​GTC​GTT​GTT​CT-3′
*IGFBP3*	FW: 5′-AGA​GCA​CAG​ATA​CCC​AGA​ACT-3′	*PRRX1*	FW: 5′-TGA​TGC​TTT​TGT​GCG​AGA​AGA-3′
	RV: 5′-GGT​GAT​TCA​GTG​TGT​CTT​CCA​TT-3′		RV: 5′-AGG​GAA​GCG​TTT​TTA​TTG​GCT-3′
*MYLPF*	FW: 5′-GAA​GGA​CAG​TAG​AGG​GCG​GAA-3′	*MYL1*	FW: 5′-GTT​GAG​GGT​CTG​CGT​GTC​TTT-3′
	RV: 5′-TCT​GGT​CGA​TCA​CAG​TGA​AGG-3′		RV: 5′-ACC​CAG​GGT​GGC​TAG​AAC​A-3′

### Flow cytometric analysis

DMD- and DMD-Iso-COs at 14 days were dissociated using Collagenase A (1 U/ml) for 20 min at 37°C followed by 10 min incubation with Accutase^®^ (Sigma-Aldrich). All flow cytometry procedures were performed according to the manufacturer’s instructions. PBS + 0.1% BSA (Sigma) was used as staining buffer. The cells were stained for the surface markers SIRPA, CD31 and PDGRFa specific for CMs, endothelial cells and fibroblast/adipose progenitors respectively. A total of 50.000 cells was recorded. After single cell gate selection, a cell number total between 15.000 and 20.000 cells was analysed for the used antibodies (CD31, PDGFRα, SIRPA). Fluorescence minus one (FMO) controls and compensations were included for appropriate gating. Samples were analysed using the FACS Canto II HTS (BD Biosciences) and the analysis was performed using FACS Diva Software. Table 2 provides a list of all flow cytometric antibodies used in this study.

**TABLE 2:** List of antibodies dilutions used for immunofluorescence analysis and flow cytometric analysis.

*Antibodies*	*Dilution*
Immunostaining
*α-Actinin (mouse, Abcam)*	1:250
*α-Sarcoglycan (mouse, Novocastra)*	1:250
β*-Sarcoglycan (mouse, Novocastra)*	1:250
γ*-Sarcoglycan (mouse, Novocastra)*	1:250
δ*-Sarcoglycan (mouse, Novocastra)*	1:250
*NKX2.5 (Rabbit, Bioke)*	1:250
*PGK1 (mouse, Santa Cruz)*	1:250
*GORASP2 (mouse, Proteintech)*	1:1000
*ARCN1 (mouse, Santa Cruz)*	1:250
*CCASP3 (Rabbit, Bioke/Cell Signaling Technology)*	1:400
*Ki67 (mouse, BD Pharmigen)*	1:300
*DYS1 (mouse, Leica)*	1:50
*DYS3 (mouse, Leica)*	1:50
*NOX4 (Rabbit, Abcam)*	1:500
Flow cytometry
*APC- anti-human CD172a SIRPA (mouse, BioLegend)*	1:100
*PE/Cy7 anti-human CD31 (mouse, BioLegend)*	1:200	
*PE anti-human CD140a PDGFRα (mouse, BD Biosciences)*	1:20	

### Immunofluorescence staining

After three PBS washes, the cryosections were permeabilized for 1 h at room temperature using 0.1% Triton X-100 in PBS (Thermo Fisher Scientific). Non-specific antibody binding was blocked by incubation for 30 min with blocking solution containing 5% normal goat serum (NGS, Dako) at room temperature followed by overnight incubation at 4°C with different primary antibodies listed in Table 2. After washing in phosphate-buffered saline (PBS), the samples were incubated with respective secondary antibodies using Alexa Fluor 488 and 555-conjugated secondary antibody (4 μg/ml; Thermo Fisher Scientific). Nuclei were counterstained with Hoechst 33342 (1:1000, Thermo Scientific) for 7 min (Santoni de Sio et al., 2008). The sections were mounted with ProLong ^™^ Gold antifade reagent (Invitrogen) and stored in the dark at 4°C until imaging. All images were acquired using Axiocam MRm microscope (Zeiss). Confocal images were acquired using Nikon ECLIPSE Ti Microscope and NIS-Elements AR 4.11 software. Quantifications of immunofluorescent images were performed using ImageJ software tool by calculating the percentage of the signal area/positive nuclei with respect to the total organoid area/total nuclei respectively for each CO.

### Quantification of beating frequency and surface area of cardiac organoids

To assess the contractile properties of DMD-COs and DMD-Iso-COs, 3D cardiac organoids were live-imaged using the Dmi1 Microscope (Leica). The recorded videos were then analysed to determine the CO beating frequency by counting the number of spontaneous contractions per minute. The cardiac organoids growth area was measured at different time points using ImageJ software tool.

### Intracellular calcium (Ca^2+^) imaging

For Ca^2+^ imaging experiments, the DMD-, DMD-Iso- and HC-hiPSC monolayers were respectively plated on 35 mm dishes with four Chamber glass bottom. Following 14 days from cardiac induction, the DMD-, DMD-Iso- and HC- CM were incubated with 1 µM Fluo-4 AM solubilized in CM Maintenance Medium. Next, the cells were washed twice with CM Maintenance Medium after which de-esterification was allowed to occur for 45 min at 37°C and 5% CO_2_. The Ca^2+^ imaging experiments were performed in pre-warmed (37°C) modified Krebs-Ringer solution (135 mM NaCl, 6.2 mM KCl, 1.2 mM MgCl_2_, 12 mM HEPES, pH 7.3, 11.5 mM glucose and 2 mM CaCl_2_). Tetracaine was solubilized in the above modified Krebs-Ringer solution at 1 mM final concentration. For the KCl stimulus the modified Krebs-Ringer solution was prepared substituting the NaCl for 140 mM KCl. Imaging was performed using a Nikon eclipse Ti2 inverted fluorescence microscope (Nikon) equipped with excitation filter FF01-378/474/554/635 and dichroic mirror FF01-432/515/595/730 and emission filter 515/30 all from Semrock. Coolled pR-4000 (Coolled) was used for excitation at 470 nm. Acquisition of the fluorescent signal at 520 nM was performed at 10 Hz using a pco.edge 4.2bi sCMOS camera (pCO) (Nakamura et al., 2001). For analysis FIJI software was utilized. In each experiment a region of interest was drawn across spontaneously active cardiomyocytes. The fluorescence intensities were normalized to F0, where the F0 value was obtained after tetracaine administration.

### RNA sequencing and bioinformatics analysis

RNA (>10 μg) extracted from DMD-COs and DMD-Iso-COs on day 56 was verified and processed by the Genomics Core (KU Leuven–UZ Leuven). As quality control, the RNA concentration was measured with Nanodrop and quality was checked with Bioanalyzer. The Lexogen QuantSeq 3′ mRNA-Seq library prep kit was used according to the manufacturer's protocol with 500 ng input. After the prep, the libraries were measured with Qubit and put on the Fragment analyser so the libraries could be pooled equimolar to 2 nM. The pool was then quantified with RT-qPCR and a final pool (2 nM) was made for single-read sequencing on the HiSeq4000 (Illumina Inc.). The settings were 51-8-8. The raw sequence files generated (.fastq files) underwent quality control analysis using FastQC v0.11.7 (Andrews, 2010). Adapters were filtered with ea-utils fastq-mcf v1.05 (Aronesty, 2011). Splice-aware alignment was performed with HiSat2 against the human reference genome hg38 using the default parameters. Reads mapping to multiple loci in the reference genome were discarded. Resulting BAM alignment files were handled with Samtools v1.5. (Li et al., 2009). Quantification of reads per gene was performed with HT-seq Count v2.7.14. Count-based differential expression analysis was done with R-based (The R Foundation for Statistical Computing, Vienna, Austria) Bioconductor package DESeq2 (Love et al., 2014). Reported *p*-values were adjusted for multiple testing with the Benjamini-Hochberg procedure, which controls false discovery rate (FDR). Gene Ontology (GO) and Biological Kyoto Encyclopedia of Genes and Genomes (KEGG) pathway enrichment analyses were identified using g:Profiler (Raudvere et al., 2019). The GO Biological Process 2018 and KEGG 2016 of each tissue were determined. The significant terms and pathways were selected with the threshold of adjusted *p*-value < 0.05. Data has been deposited in the NCBI Gene Expression Omnibus (GEO) repository under accession code GSE194297.

### Generation of protein-protein interaction (PPI) network

The PPI network of differentially upregulated genes in DMD-COs was constructed by feeding a list of gene symbols and their log_2_fold changes into the NetworkAnalyst platform (http://www.networkanalyst.ca/) using the IMEx interactome database with Steiner Forest Network (SFN) reduction algorithm. Subsequently, the gene-miRNA interactions (Rotini et al., 2018) for the selected KEGG pathways were constructed based on the miRTarBase (v8.0) database, and the network was reduced using the SFN algorithm. The degree of each node was calculated based on its number of connections to other nodes. In the network, the area of an individual node indicates the degree, and the color represents the expression. The identified top five miRNAs were mapped out in the KEGG pathways to show their interactions with the genes of a particular pathway.

### Statistical analysis

Data were statistically analysed using GraphPad Prism. All data were reported as mean ± standard deviation (SD). Differences between groups were examined for statistical significance using ANOVA and two-way ANOVA. Significance of the differences was indicated as follows: *p < 0.05, **p < 0.01, ***p < 0.001, and ****p < 0.0001.

## Results

### Characterization of the generated cardiomyocytes monolayers from DMD- and isogenic corrected hiPSC lines

**FIGURE 1:**
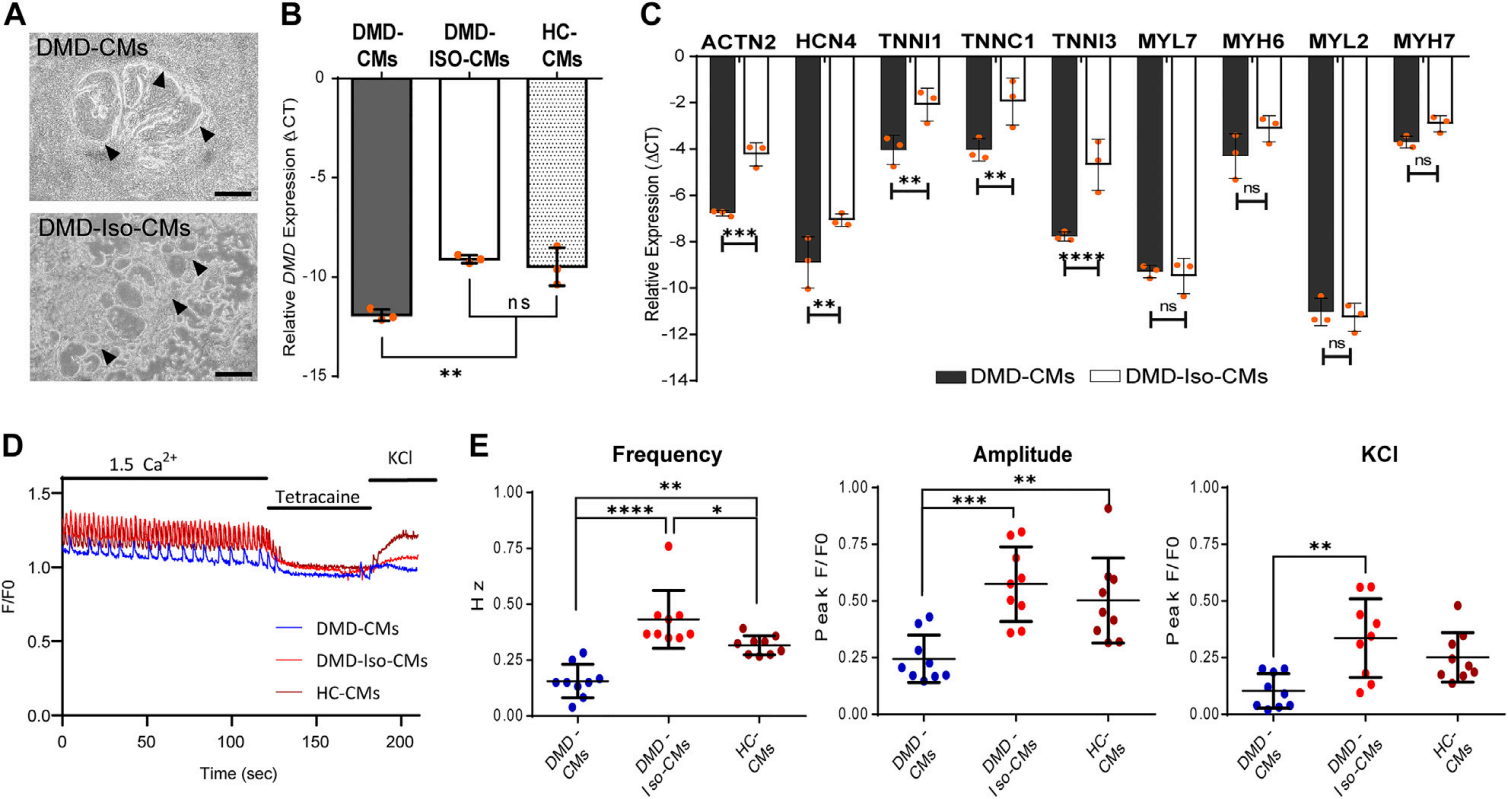
**(A)** Representative 2D culture morphology of differentiated cardiomyocytes from DMD patient-derived hiPSCs (DMD-CMs) and the isogenic controls (DMD-Iso-CMs) on day 22. Arrowheads indicated the contractile filaments. Scale bar = 200 μm. **(B**,**C)** Comparison of the expression of *dystrophin* (*DMD*) and key cardiac gene markers between DMD-CMs, DMD-Iso-CMs and HC-CMs 2D cultures on day 22. Data shown are mean ± s.d. (*n* = 3). Statistical analyses were performed by two-way ANOVA with Tukey’s multiple comparisons: **p < 0.01, ***p < 0.001, ****p < 0.0001, n.s, not significant. **(D**,**E)** Intracellular Ca^2+^ imaging of DMD-CMs, DMD-Iso-CMs and HC-CMs 2D cultures on day 14 showing higher frequency and amplitude of spontaneous Ca^2+^ oscillation in DMD-CMs than the isogenic and healty controls, as well as a lower KCl response compared to DMD-Iso-CMs. Tetracaine was used to validate that Ca^2+^ oscillations were driven by RyR channels (*n* = 9). Statistical analyses were performed by two-way ANOVA with Tukey’s multiple comparisons: *p < 0.05, **p < 0.01, ***p < 0.001, ****p < 0.0001.

Following the 2D monolayer differentiation protocol, we generated cardiomyocytes (CMs) from both DMD patient-derived hiPSC (DMD-CMs), the isogenic control hiPSC (DMD-Iso-CMs) and healthy control hiPSC (HC-CMs) monolayer cultures. Figure 1A shows representative images of 2D culture morphology of DMD-CMs and DMD-Iso-CMs on day 22. These cells started to develop contractile phenotype around day 8 and were morphologically similar in both conditions. On day 22, RT-qPCR analysis showed that the DMD-CMs expressed significantly lower dystrophin (*DMD*) than the isogenic controls and HC-CMs (Figure 1B), confirming the restoration of *DMD* expression in the isogenic controls, as described in Duelen et al. (2022). The DMD-CMs also expressed significantly lower sarcomeric α-actinin (*ACTN2*), the pacemaker gene *HCN4*, and the troponin-related genes (*TNNI1, TNNC1* and *TNNI3*) but not the myosin light (*MYL7*, *MYL2*) or heavy chain (*MYH6*, *MYH7*) genes, than the isogenic controls (Figure 1C). Next, we established a cell physiological analysis of 14-day differentiated DMD-CMs and DMD-Iso-CMs. A hallmark of functional CMs is their ability to generate cytosolic Ca^2+^ signals that are driven by ryanodine receptors (RyRs), intracellular Ca^2+^ -release channels residing at the sarcoplasmic reticulum of CMs. Therefore, cytosolic Ca^2+^ imaging was performed in single-cell CMs loaded with Fluo-4. In the presence of extracellular Ca^2+^ (1.5 mM CaCl_2_), spontaneous Ca^2+^ oscillations were observed in both DMD-CMs and DMD-Iso-CMs that could be blocked by tetracaine, an inhibitor of RyR channels. However, spontaneous Ca^2+^ oscillations appeared to display a lower frequency and amplitudes in DMD-CMs compared to isogenic and healthy controls, indicating a defect in physiological Ca^2+^ signalling in dystrophic CMs that is corrected in the isogenic controls. Moreover, DMD-CMs displayed a lower Ca^2+^ response to KCl, which provokes membrane depolarization, compared to DMD-Iso-CMs (Figures 1D,E). These findings validated the dystrophic properties of DMD-CMs and their defects in physiological Ca^2+^ signalling, whereby both deficiencies could be reverted in DMD-Iso-CMs generated in this study.

### Generation of DMD- and DMD-isogenic corrected cardiac organoids (COs)

**FIGURE 2:**
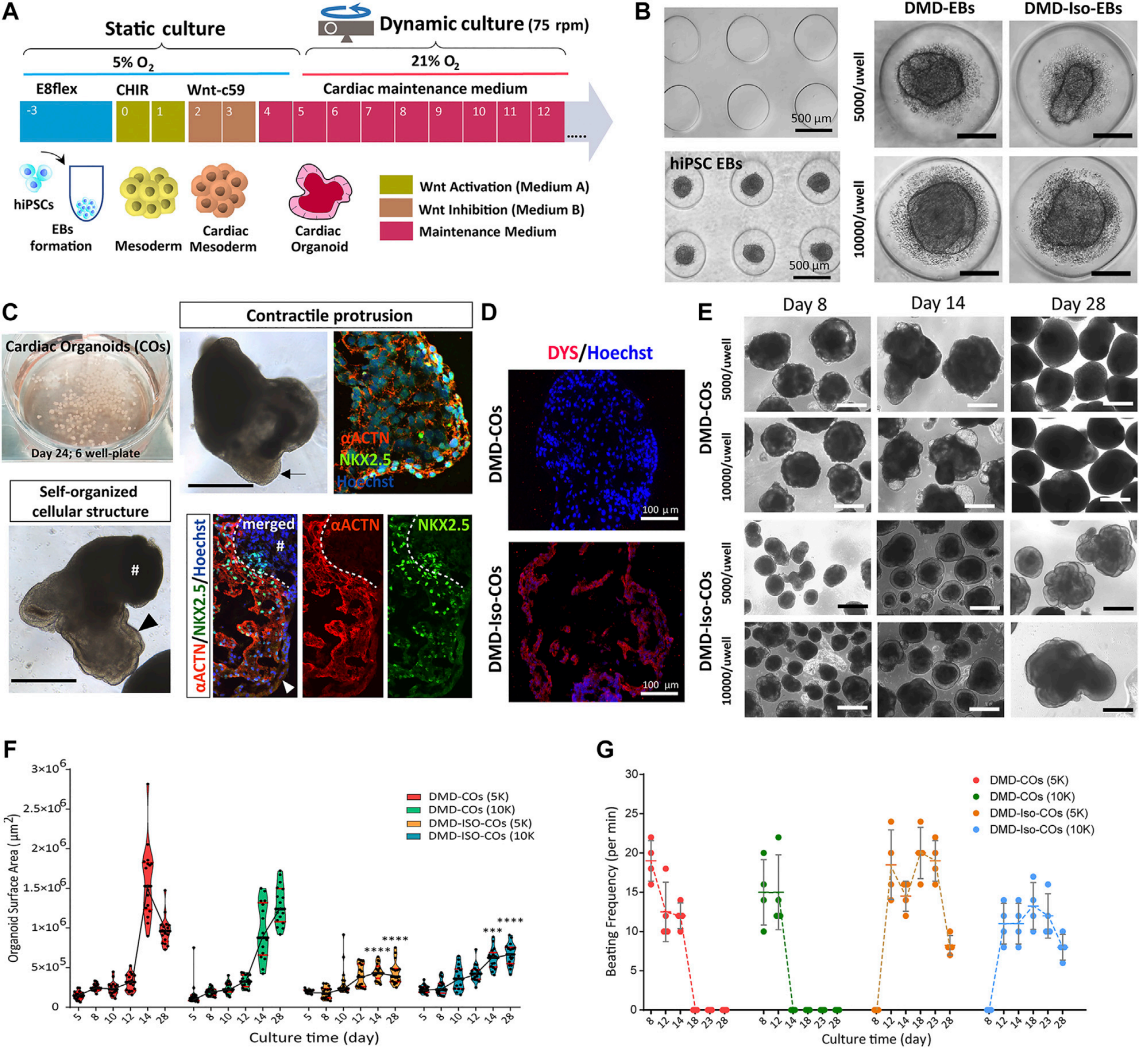
Generation of DMD-COs and DMD-Iso-COs from patient-derived hiPSC. **(A)** Schematic showing the culture protocol to generate DMD-COs and DMD-Iso-COs from patient-derived hiPSC embryoid bodies (EBs). **(B)** Brightfield images of the microwells of the agarose insert and the formed EBs. **(C)** EBs pooled from two agarose inserts inside a well of six-well plate for dynamic culture, and the morphology of COs at high magnification showing contractile cardiomyocyte protrusion (arrow) and distinct self-organized cellular structure at the organoid periphery (arrowhead) both with specific spatial distribution of NKX2.5 and αACTN positivity. Non-translucent organoid structure (#) was negative for NKX2.5 and αACTN. **(D)** Immunofluorescence staining showing the localization of DYS in DMD-Iso-COs, which was undetectable in DMD-COs. Nuclei were counterstained with Hoechst. **(E)** Representative images of CO morphology and the changes of organoid size (generated at two cells seeding numbers) over 28 days of dynamic culture. **(F**,**G)** Effect of cell seeding numbers on the growth (*n* = 17) and the beating frequency (*n* = 4) of DMD-COs or DMD-Iso-COs over 28 days. DMD-COs versus DMD-Iso-COs at 5K (5,000 cells/microwell) or 10K (10,000 cells/microwell). Statistical analyses were performed by two-way ANOVA with Tukey’s multiple comparisons: ***p < 0.001, ****p < 0.0001. CO, Cardiac organoid; DMD-COs and DMD-Iso-COs, cardiac organoids from DMD patient-derived hiPSC and isogenic corrected hiPSC, respectively. Scale bar = 1 mm or as stated in the figure.

We adapted the cardiomyocyte monolayer differentiation protocol to generate COs by direct differentiation of the embryoid bodies (EBs) (Figure 2A). By using the agarose microwell culture inserts, we could promote self-aggregation of the DMD-hiPSCs and DMD-Iso-hiPSCs into EBs at cell seeding number of 5,000 and 10,000 cells per microwell (Figure 2B). This allowed us to generate 137 EBs per insert per well of 24-well plate. On day 5 of cardiomyocyte differentiation, the resulting DMD-COs and DMD-Iso-COs were transferred to 6-well plate on orbital shaker for dynamic culture in the CM maintenance medium (Figure 2C). Contractile cardiomyocyte protrusions (Figure 2C, arrow) and self-organized cellular structures (Figure 2C, arrowheads) at the organoid periphery, both with specific spatial distribution of NKX2.5 and αACTN positivity, could be observed. The non-translucent organoid structure (#) was negative for both NKX2.5 and αACTN. Immunofluorescence staining showed abundant DYS localization in DMD-Iso-COs, which was undetectable in the DMD-COs (Figure 2D). The representative images of CO over 28 days of dynamic culture showed morphological changes and variations in organoid size (Figure 2E). Quantification of the organoid surface area over 28 days of dynamic culture showed no significant differences on the organoid size between the two cell seeding numbers within each cell line, however, the size of DMD-Iso-COs was significantly smaller than DMD-COs on day 14 and 28, respectively (Figure 2F). The DMD-COs displayed contraction on day 8 (± 19 per minute) which decreased over time and stopped contraction between day 14 and 18 (Figure 2G). The DMD-Iso-COs displayed contraction on day 12 (± 18 per minute) which persisted till day 28. FACS analysis was performed on DMD- and DMD-Iso-COs after 14 days to determine SIRPA (APC), CD31 (PE/Cy7) and PDGFRα (PE) positive cells (Supplementary Figures S1A,B). The analysis disclosed that both DMD- and DMD-Iso-COs accounted for 40/50% of SIRPA^+^ cells, while DMD-Iso-COs showed the double amount of the CD31^+^ cell (1.4% ± 0.2) population and the half of the PDGFRα^+^ cells (1.7% ± 0.2) compared to DMD-COs.

### DMD-CO and DMD-Iso-CO characterization and progressive loss of sarcoglycans in DMD-COs

**FIGURE 3:**
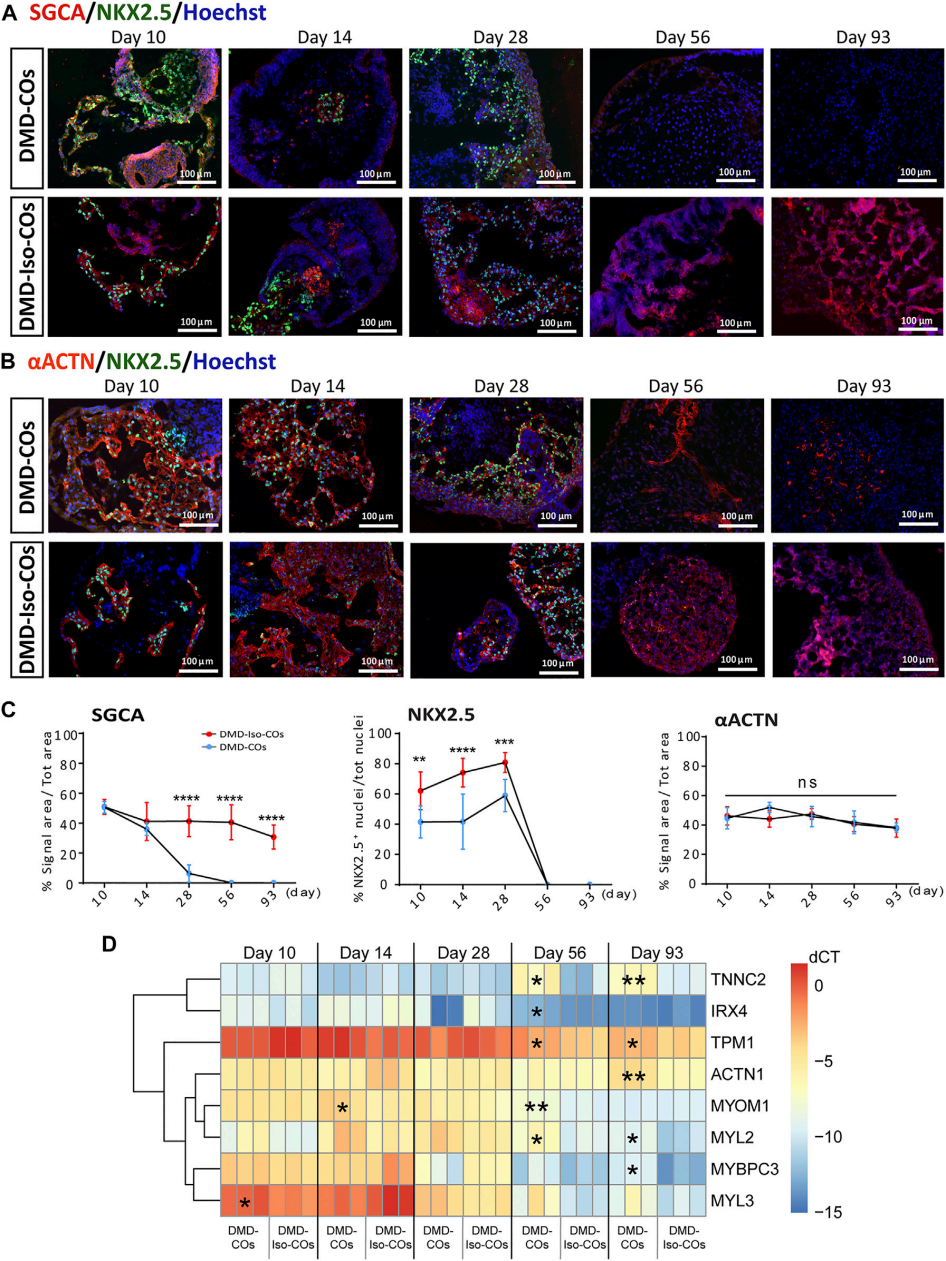
Assessment of cardiac differentiation and contractile proteins development in DMD-COs and DMD-Iso-COs over 93 days of dynamic culture. Representative immunofluorescence images for: **(A)** α-sarcoglycan (SCGA)/NKX2.5, and **(B)** sarcomeric α-actinin (αACTN)/NKX2.5 co-staining in DMD-COs and DMD-Iso-COs, respectively. Nuclei were counterstained with Hoechst. Data are representative of three independent experiments (*n* = 3). (Magnification: ×20). **(C)**Quantification of the immunofluorescence images for: SGCA, NKX2.5 and αACTN on day 10, 14 28, 56 and 93. Data shown are mean ± s.d. (*n* = 4, two-way ANOVA with Sidak’s multiple comparisons: *p < 0.05, **p < 0.01, ***p < 0.001, ****p < 0.0001. **(D)** RT-qPCR analysis of representative gene markers expression for cardiac contractility in DMD-COs and DMD-Iso-COs. Data shown are mean ± s.d. (*n* = 3, each pooled from ∼10 organoids). Statistical analyses were performed by two-way ANOVA with Tukey’s multiple comparisons: *p < 0.05, **p < 0.01.

We performed immunofluorescence staining for α-sarcoglycan (SCGA), sarcomeric α-actinin (αACTN), and NKX2.5 on day 10, 14 28, 56 and 93 in order to assess cardiac differentiation and contractile protein development within the organoids. The results showed abundant SCGA expression in DMD-COs on day 10, which became low on day 14 and undetectable from day 28 onwards (Figures 3A,C and Supplementary Figure S2A). Conversely, the SCGA expression in DMD-Iso-COs persisted till day 93. As reported in Supplementary Figure S3A also the β-, γ- and δ-sarcoglycans started to disappear from day 14 following the α-sarcoglycan trend. A transient expression of the early cardiac differentiation marker NKX2.5 was observed up to day 28 in both DMD-COs and DMD-Iso-COs, which became undetectable on day 56 and 93 (Figure 3C). Additionally, abundant αACTN, a cardiac contractile protein, was observed in both DMD-COs and DMD-Iso-COs on early time points, which remained detectable on day 93 (despite at lower expression level) in both conditions (Figures 3B,C and Supplementary Figure S2B). There was no distinguishable difference in the SCGA, αACTN and NKX2.5 expression between organoids generated from the two cell seeding numbers within a cell line. These results demonstrated a progressive loss of SCGA protein expression in DMD-COs (a member of the dystrophin associated complex, DAC) as compared to the isogenic controls. The sarcomeric pattern is shown at higher resolution and magnification in Supplementary Figure S3B which reports representative confocal images of αACTN and SCGA*.* In order to better characterize the cardiomyocyte population within the COs we performed the RT-qPCR for atrial/ventricular gene markers and TNNT2 at all the time points (Supplementary Figures S4A,B). Both atrial and ventricular markers showed a peak at the initial stage followed by a decline at later stages. Intriguingly IRX4, a ventricle-specific transcription factor, appeared to be always expressed in both samples until day 28 and declined at late stages as the majority as the other atrial and ventricle markers. However, the two major ventricular myosin heavy-chain genes MYL2 and MYH7 were sustained longer during the differentiation compared to the atrial ones (Supplementary Figure S4A). Likewise, TNNT2 expression reached the peak at day 14 and then decreased without showing striking differences between the two cell lines (Supplementary Figure S4B). Additionally, RT-qPCR analysis showed a significant upregulation of genes related to cardiac contractility in DMD-COs as compared to DMD-Iso-COs, in particularly from day 56 onwards (Figure 3D). These included *ACTN1*, *IRX4*, *MYBPC3*, *MYL2*, *MYOM1*, *TNNC2* and *TPM1*.

### Lack of initial proliferative capacity, high levels of NADPH oxidase 4 (NOX4) and endoplasmic reticulum stress markers in DMD-COs

**FIGURE 4:**
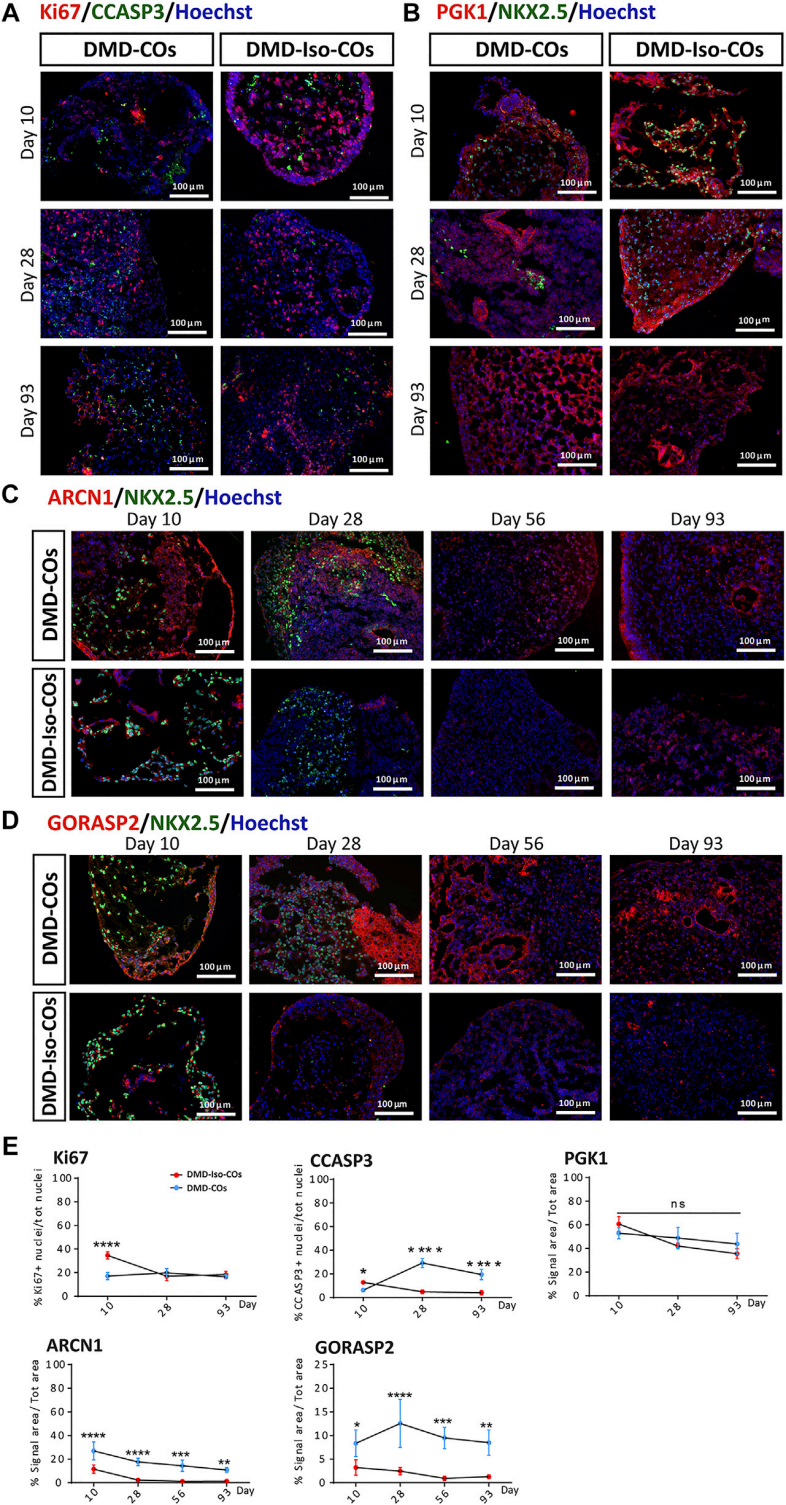
Assessment of cell proliferation, apoptosis and ER stress in DMD-COs and DMD-Iso-COs over 93 days of dynamic culture. Representative immunofluorescence images for: **(A)** Ki67/CCASP3, **(B)** PGK1/NKX2.5 on day 10, 28 and 93; **(C)** ARCN1/NKX2.5 and **(D)** GORASP2/NKX2.5 on day 10, 28, 56 and 93. Nuclei were counterstained with Hoechst. Data are representative of three independent experiments (*n* = 3). (Magnification: ×20). **(E)** Quantification of the immunofluorescence images for: Ki67, CCASP3 and PGK1 on day 10, 28 and 93; ARCN1 and GORASP2 on day 10, 28, 56 and 93. Data shown are mean ± s.d. (*n* = 4, two-way ANOVA with Sidak’s multiple comparisons: *p < 0.05, **p < 0.01, ***p < 0.001, ****p < 0.0001.

We examined cell proliferation and apoptotic conditions within the DMD-COs and DMD-Iso-COs by immunostaining of the proliferation marker Ki67 and apoptotic marker cleaved caspase 3 (CCASP3). The results showed low Ki67 staining in DMD-COs but relatively higher signal in DMD-Iso-COs on day 10, while the signal became comparable on day 28 and 93 (Figures 4A,E and Supplementary Figure S2C). Though initially CCASP3 signal area was slightly more in DMD-Iso-COs, from day 28 it became significantly higher in DMD-COs (Figure 4E). However, no significant difference in Ki67 and CCASP3 staining was observed at both cell seeding densities for both COs conditions (data not shown). These data suggest that the DMD-COs was lacking an initial proliferative capacity at early time point while at later time points, they were more apoptotic. We then assessed the metabolic activity within the COs by immunostaining of the glycolytic marker phosphoglycerate kinase 1 (PGK1). The results showed high and comparable PGK1 staining in both COs conditions at all time points (Figures 4B,E and Supplementary Figure S2D), which was independent from the cell seeding densities (data not shown). These results suggest the glycolytic condition of immature COs in both CO conditions. The cellular stress was assessed by immunostaining of two known endoplasmic reticulum (ER) stress markers ARCN1 and GORASP2. Interestingly, we detected relatively higher level of ARCN1 in DMD-COs than DMD-Iso-COs at all the time points (Figures 4C,E and Supplementary Figure S2E), whereas GORASP2 increased progressively over the 28 days in DMD-COs, independently from the cell seeding densities (data not shown) and at higher level than that in DMD-Iso-COs at all the time points (Figures 4D,E and Supplementary Figure S2F). This finding indicated a high level of ER stress occurred in DMD-COs. To assess oxidative stress due to high NOX4 expression, we performed immunofluorescence staining on the organoid cryosections for NOX4. As shown in Supplementary Figure S5A, high NOX4 protein level was detected in the DMD-COs on day 14 which was barely present in DMD-Iso-COs.

### Progressive cardiac phenotype deterioration in DMD-CO long term culture

**FIGURE 5:**
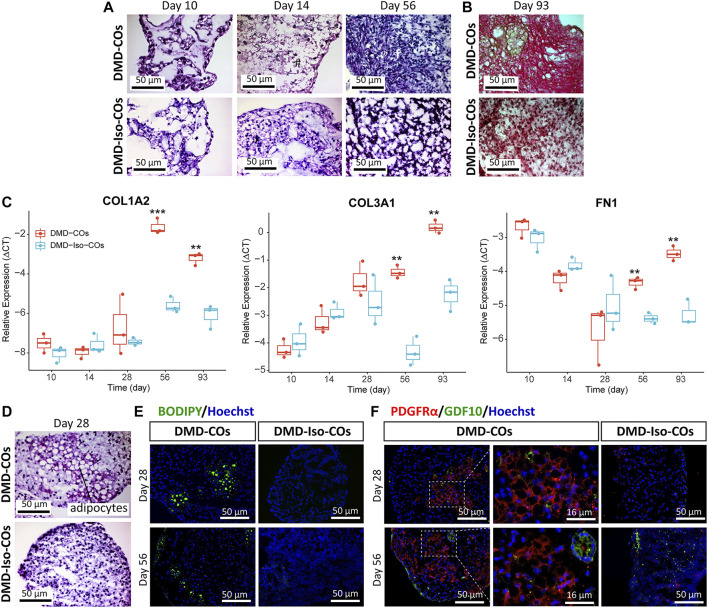
Assessment of fibrosis and adipogenesis in DMD-COs and DMD-Iso-COs over 93 days of dynamic culture. **(A)** H&E staining showing deterioration of cardiomyocyte tissue on day 14 and fibrosis on day 56 in DMD-COs. (Magnification: ×40). **(B)** Picro-Sirius red staining showing abundant collagen deposition in DMD-COs on day 93. (Magnification: ×40). **(C)** RT-qPCR analysis of representative fibrosis gene markers showing a significant upregulation of *COL1A2*, *COL3A1* and *FN1* expression in DMD-COs on day 56 and 93 as compared to DMD-Iso-COs. Data shown are mean ± s.d. (*n* = 3, each pooled from ∼10 organoids). Statistical analyses were performed by two-way ANOVA with Tukey’s multiple comparisons: **p < 0.01, ***p < 0.001. **(D**–**F)** Adipogenesis in DMD-COs as indicated by the formation of adipocytes with cytoplasmic vacuoles (H&E staining), lipid droplet deposition (BODIPY staining), and PDGFRα positivity on day 28 and 56. The adipogenesis inhibitor GDF10 was also detected near the PDGFRα^+^ cells in DMD-COs. Nuclei were counterstained with Hoechst. (Magnification: ×20 and ×40). Data are representative of three independent experiments (*n* = 3).

We performed histological examination to assess any cytoarchitecture changes and DMD-related pathological progression within the COs over 93 days. The DMD-COs displayed normal cardiomyocyte-like structures similar to that of DMD-Iso-COs on day 10, which deteriorated on day 14 (indicated as “#”) and developed fibrotic-like structures (indicated as “f”) at later time points (Figure 5A; *H&E staining* on day 56, and Figure 5B; Picro-Sirius red staining for collagen deposition on day 93). These findings were corroborated by a significant upregulation of gene markers associated with fibrosis *COL1A2*, *COL3A1* and *FN1* in DMD-COs on day 56 and 93 as compared to DMD-Iso-COs (Figure 5C). Additionally, H&E staining also revealed adipose tissue formation in DMD-COs on day 28 (Figure 5D), confirmed by the detection of lipid droplets *via* BODIPY staining and immunolabelled PDGFRα^+^ cells (an adipocyte marker) in DMD-COs on day 28 and 56 (Figure 5E). Interestingly, GDF10 protein (an adipogenesis inhibitor) was also detected near the PDGFRα^+^ cells in DMD-COs (Figure 5F). These findings suggest that DMD-COs displayed an initial normal cardiac phenotype which deteriorated progressively and exhibited pro-fibrotic and adipogenic phenotypes upon long-term culture, resembling pathologic events associated with DMD cardiomyopathy.

### RNA sequencing revealed functional enrichment of hypertrophy/dilated cardiomyopathy, adipogenesis and fibrosis signalings in DMD-COs

**FIGURE 6:**
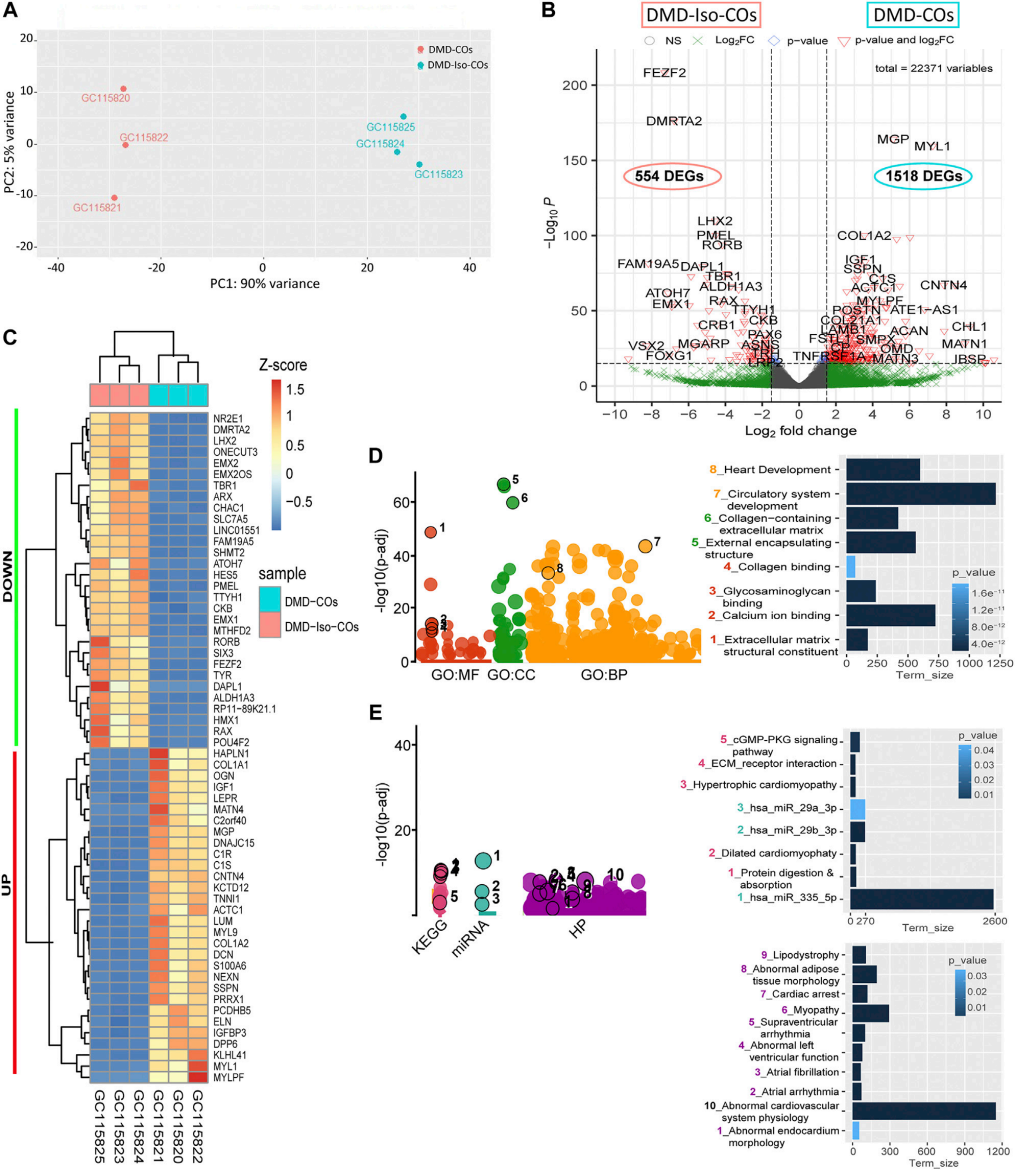
RNA sequencing analysis of DMD-COs and DMD-Iso-COs *via* DESeq2 method. **(A)** Principal component analysis (PCA) showing distinct separation of the DMD-COs and DMD-Iso-COs clusters (PC1: 90%) with low intra-condition variance (PC2: 5%) in both conditions, respectively. **(B)** Enhanced volcano plot showing the differentially expressed genes (DEGs) in DMD-COs versus DMD-Iso-COs. Cut-off log_2_ fold change = 1.5; Cut-off −Log_10_ *p* = 10^–16^. **(C)** Heatmap showing the TOP30 DEGs in DMD-COs versus DMD-Iso-COs. **(D**,**E)** Functional enrichment analysis of the differentially upregulated genes in DMD-COs versus DMD-Iso-COs using gProfiler2 for Gene ontology, KEGG pathway, miRNA and human phenotype ontology. (All data shown: *n* = 3, each sample was a pooled of ∼10 organoids).

Principal component analysis of the RNA transcriptomic data showed a distinct separation between DMD-COs and DMD-Iso-COs clusters (PC1: 90%) with low intra-condition variance (PC2: 5%) (Figure 6A). Based on the enhanced volcano plot, out of 22,371 gene variables, 1,518 and 554 genes were differentially upregulated in DMD-COs and DMD-Iso-COs, respectively (Cut-off: log_2_ fold change = 1.5; −Log_10_ *p* = 10^–16^) (Figure 6B). Among the top 30 most differentially upregulated genes in DMD-COs (Figure 6C), *MGP*, *MYL1*, *COL1A2*, *HAPLN1* and *OGN* were the five most significant upregulated genes (Figure 6B and Table 3). In addition, we validated the upregulation of the majority of the genes in DMD-COs showed in Figure 6B by RT-qPCR in DMD-, DMD-Iso- and HC-COs at day 56 as additional control (Supplementary Figure S6A). Based on gProfiler analysis, gene ontologies that were significantly enriched for molecular function in extracellular matrix regulation (i.e. collagen and glycosaminoglycan; *GO:MF*), cardiac tissue structure formation (i.e. external encapsulating structure such as sarcolemma; *GO:MM*), and cardiovascular development (*GO: BP*) could be identified in DMD-COs (Figure 6D). Additionally, KEGG pathways associated with protein digestion and absorption, dilated and hypertrophic cardiomyopathy, ECM-receptor interaction, and cGMP-PKG signalling pathway (known to positively modulates cardiac contractility, hypertrophy and protects against apoptosis (Takimoto, 2012) were significantly enriched in DMD-COs (Table 4 and Figure 6E (i)). These findings were corroborated by the analysis on human phenotype ontology, whereby ontology related to abnormal cardiovascular system physiology, including abnormal left ventricular function, abnormal endocardium morphology, atrial arrhythmia and fibrillation, supraventricular arrhythmia, myopathy and cardiac arrest, as well as abnormal adipose tissue morphology and lipodystrophy were significantly enriched in DMD-COs as compared to DMD-Iso-COs (Figure 6E (ii)). Moreover, the gProfiler analysis also identified three top miRNA regulators for the differentially upregulated genes in DMD-COs, namely *hsa-mir-335-5p*, *hsa-mir-29a-3p* and *hsa-mir-29b-3p*. Altogether, the RNA sequencing data validated the histological observations described above on cardiomyocyte deterioration, adipogenesis and fibrosis at the transcriptomic level.

**TABLE 3:** Top30 differentially upregulated and down-regulated genes in DMD-COs versus DMD-Iso-COs.

	ID	Gene name	baseMean	log2FoldChange	lfcSE	stat	pvalue	Padj
Differentially Upregulated Genes	13088	MGP	3880.521907	5.120819425	0.187135	27.36434	7.29E−165	5.44E−161
	13862	MYL1	2675.966929	7.262145462	0.269213	26.9755	2.87E−160	1.60E−156
	4878	COL1A2	14733.15743	3.549526614	0.166558	21.31101	8.97E−101	2.87E−97
	9557	HAPLN1	1152.713973	6.021041431	0.284317	21.17721	1.55E−99	4.33E−96
	14864	OGN	1806.551067	5.301108486	0.251858	21.04799	2.39E−98	5.93E−95
	10461	IGF1	2063.104984	3.348551855	0.171565	19.51771	7.76E−85	1.58E−81
	3270	C1R	836.7627027	3.594911367	0.186023	19.32509	3.30E−83	6.16E−80
	6414	DCN	6535.506649	3.199249115	0.168451	18.99221	1.98E−80	3.16E−77
	26211	SSPN	1223.773057	3.454929985	0.184017	18.77501	1.21E−78	1.69E−75
	10472	IGFBP3	2242.532837	3.187765804	0.174276	18.29151	9.67E−75	1.14E−71
	12548	LUM	1859.659818	3.902455102	0.213459	18.28201	1.15E−74	1.29E−71
	3273	C1S	505.5575661	4.532842321	0.25232	17.96466	3.69E−72	3.44E−69
	16517	PRRX1	2296.492692	3.165414194	0.176446	17.93981	5.77E−72	5.16E−69
	13878	MYL9	2014.301562	3.047364639	0.171162	17.80398	6.58E−71	5.66E−68
	4832	CNTN4	438.9813428	7.862840625	0.451874	17.40051	8.18E−68	6.53E−65
	6893	DPP6	575.4551543	8.475259781	0.487454	17.3868	1.04E−67	8.01E−65
	12815	MATN4	464.8395192	5.461265657	0.315708	17.29848	4.83E−67	3.60E−64
	1178	ACTC1	2989.236594	4.07554558	0.236959	17.19934	2.69E−66	1.88E−63
	4877	COL1A1	17931.90071	3.760557058	0.221112	17.00747	7.23E−65	4.90E−62
	15297	PCDHB5	332.9803767	4.835046959	0.285038	16.96279	1.55E−64	9.90E−62
	24364	S100A6	1991.282073	3.116793963	0.185219	16.82763	1.53E−63	9.51E−61
	11066	KCTD12	2475.653645	2.587393647	0.153899	16.81233	1.98E−63	1.20E−60
	13886	MYLPF	895.0795595	4.39539839	0.275249	15.96882	2.11E−57	1.18E−54
	27539	TNNI1	1506.276513	3.591374007	0.225007	15.96117	2.38E−57	1.30E−54
	11676	LEPR	1025.641125	3.609301571	0.229042	15.75825	6.03E−56	3.14E−53
	6784	DNAJC15	250.7545201	4.796254742	0.305622	15.69341	1.68E−55	8.53E−53
	3362	C2orf40	1709.870968	2.953046306	0.189313	15.59879	7.42E−55	3.53E−52
	11319	KLHL41	358.1653311	4.180634798	0.268513	15.56955	1.17E−54	5.46E−52
	7375	ELN	3805.846419	2.592306878	0.16688	15.534	2.04E−54	9.32E−52
	14268	NEXN	1403.167628	3.120924866	0.201871	15.45996	6.46E−54	2.84E−51
Differentially Down-regulated Genes	8257	FEZF2	2147.508792	−7.297943854	0.236509	−30.857	4.52E−209	1.01E−204
	6732	DMRTA2	1410.513095	−6.781574008	0.239105	−28.3624	5.89E−177	6.59E−173
	11722	LHX2	3925.790741	−4.554419135	0.20393	−22.3332	1.76E−110	7.87E−107
	15965	PMEL	1206.380481	−4.518113856	0.211711	−21.341	4.73E−101	1.76E−97
	18101	RORB	4525.902649	−4.172628967	0.20232	−20.6239	1.67E−94	3.74E−91
	7899	FAM19A5	746.1460517	−8.183408649	0.426594	−19.1831	5.12E−82	8.81E−79
	6352	DAPL1	1233.729736	−5.235519232	0.276173	−18.9574	3.84E−80	5.72E−77
	7421	EMX2	2184.20914	−3.94754281	0.214817	−18.3763	2.03E−75	2.68E−72
	7422	EMX2OS	843.8705185	−3.862243505	0.210282	−18.367	2.41E−75	3.00E−72
	26756	TBR1	1184.062948	−4.109792654	0.226273	−18.163	1.01E−73	1.08E−70
	9992	HMX1	602.267519	−5.86682695	0.323561	−18.132	1.78E−73	1.81E−70
	28131	TYR	391.1820556	−4.982659101	0.275952	−18.0563	7.04E−73	6.85E−70
	2364	ARX	634.2942288	−5.00821928	0.286738	−17.4662	2.59E−68	2.15E−65
	1658	ALDH1A3	643.2412907	−3.696045432	0.213864	−17.2822	6.40E−67	4.62E−64
	25017	SIX3	1428.324473	−3.282550679	0.193132	−16.9964	8.73E−65	5.74E−62
	2492	ATOH7	469.676258	−7.115070692	0.426177	−16.6951	1.42E−62	8.37E−60
	17083	RAX	577.8533812	−4.099396951	0.256498	−15.9822	1.70E−57	9.75E−55
	9702	HES5	1171.343235	−2.936085758	0.184229	−15.9371	3.50E−57	1.86E−54
	7420	EMX1	323.5042159	−6.926075308	0.44215	−15.6645	2.64E−55	1.31E−52
	22627	RP11-89K21.1	382.2280006	−4.214816465	0.269141	−15.6603	2.83E−55	1.37E−52
	14893	ONECUT3	285.5907893	−5.959062474	0.384535	−15.4968	3.65E−54	1.63E−51
	12209	LINC01551	307.298716	−6.914384951	0.44911	−15.3957	1.75E−53	7.52E−51
	28033	TTYH1	2791.568121	−2.459452396	0.162906	−15.0974	1.68E−51	6.61E−49
	14611	NR2E1	319.4212395	−4.893422773	0.326486	−14.9882	8.77E−51	3.17E−48
	4464	CHAC1	995.4565501	−2.920428254	0.195376	−14.9477	1.61E−50	5.72E−48
	25400	SLC7A5	861.7666728	−2.941521316	0.200486	−14.6719	9.75E−49	3.16E−46
	16129	POU4F2	369.4507945	−3.970980557	0.271888	−14.6052	2.60E−48	8.20E−46
	13703	MTHFD2	1961.02617	−2.063767517	0.141852	−14.5487	5.95E−48	1.82E−45
	24961	SHMT2	3444.426553	−1.989370244	0.137842	−14.4322	3.25E−47	9.81E−45
	4621	CKB	15898.89106	−1.934043819	0.138295	−13.9849	1.93E−44	5.32E−42

**TABLE 4:** List of identified TOP20 KEGG pathways based on the upregulated DEGs in DMD-COs (Cut-off log2FC > 1.5, Cut-off *p*-value <0.05).

Pathway	Total	Expected	Hits	*p* Value	FDR
ECM-receptor interaction	82	8.64	37	8.86E−16	2.82E−13
Protein digestion and absorption	90	9.48	36	2.06E−13	3.27E−11
Complement and coagulation cascades	79	8.32	33	4.89E−13	4.27E−11
Focal adhesion	199	21	57	5.37E−13	4.27E−11
PI3K-Akt signaling pathway	354	37.3	75	1.29E−09	8.18E−08
Hypertrophic cardiomyopathy (HCM)	85	8.95	27	7.44E−08	3.94E−06
Dilated cardiomyopathy	91	9.59	27	3.56E−07	1.62E−05
Amoebiasis	96	10.1	27	1.15E−06	4.59E−05
Calcium signaling pathway	188	19.8	42	1.48E−06	5.23E−05
Renin secretion	69	7.27	20	1.72E−05	0.000546
Proteoglycans in cancer	201	21.2	41	2.11E−05	0.000611
Pathways in cancer	530	55.8	85	3.49E−05	0.000926
Regulation of actin cytoskeleton	214	22.5	42	4.39E−05	0.00107
PPAR signaling pathway	74	7.8	20	5.25E−05	0.00119
cGMP-PKG signaling pathway	166	17.5	34	9.70E−05	0.00206
Cytokine-cytokine receptor interaction	294	31	52	0.000106	0.0021
Arrhythmogenic right ventricular cardiomyopathy (ARVC)	72	7.58	19	0.000114	0.00214
Retinol metabolism	67	7.06	18	0.000134	0.00237
Maturity onset diabetes of the young	26	2.74	10	0.000175	0.00293
Vascular smooth muscle contraction	132	13.9	28	0.000212	0.00336

### Protein-protein interaction (PPI) network analysis of differentially upregulated genes in DMD-COs

**FIGURE 7:**
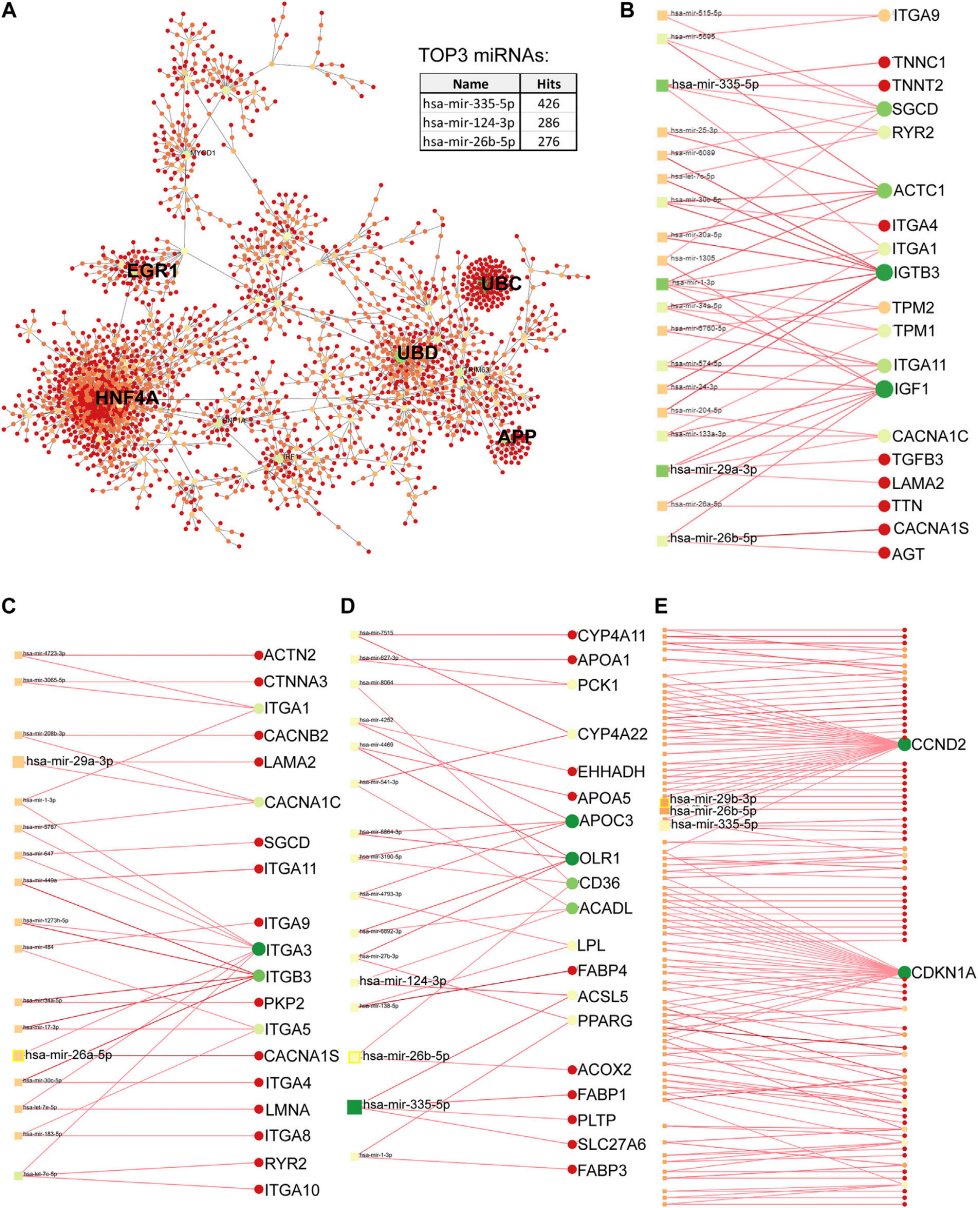
Protein-protein interaction network analysis based on differentially upregulated genes in DMD-COs using Network Analyst platform. **(A)** Top five main hub genes (HNF4A, UBC, UBD, APP and EGR1) were identified based on the degree levels. The gene-miRNA networks for hypertrophy/dilated cardiomyopathy **(B)**, arrhythmogenic right ventricular cardiomyopathy **(C)**, PPARgamma **(D)** and PI3K-Akt **(E)** signalling pathways, respectively. The colours indicate the degree of interaction (with lowest to highest degree from red to yellow to green), whereas the sizes of circles indicate the degree of betweenness of a node (larger size means higher degree of betweenness). The identified top three miRNAs by PPI and top two miRNAs by gProfiler2 analysis were mapped in each gene-miRNA network to indicate the genes they interact with.

PPI analysis of the differentially upregulated genes in DMD-COs revealed a gene network consisted of 2,289 nodes and 2,288 edges. According to the degree level (d), the top five hub nodes were HNF4A (d = 257), UBC (d = 108), UBD (d = 66), APP (d = 38) and EGR1 (d = 31) (Figure 7A). By exploring the miRNA database (i.e. mirTarBase v8.0), the top three miRNA regulators of this gene network were *hsa-mir-335-5p*, *hsa-mir-124-3p*, and *hsa-mir-26b-5p*. Together with the *hsa-mir-29b-3p* and *hsa-mir-29a-3p* identified by gProfiler2, we mapped out these miRNAs on the gene-miRNA regulatory networks for the selected KEGG pathways relevant to the DMD-COs phenotypes: *1*) Hypertrophy cardiomyopathy, *2*) Dilated cardiomyopathy, *3*) Arrhythmogenic right ventricular cardiomyopathy (ARVC), 4) PPAR signalling pathway (for adipogenesis), and 5) PI3K-Akt signalling pathway (for cardiac fibrosis (Table 5) (Qin et al., 2021)). The results showed that hypertrophy and dilated cardiomyopathy networks shared the same gene set (50 nodes, 49 edges), miRNA interactions (Figure 7B) and 16 genes similarity with the ARVC network (Figure 7C). Except *hsa-mir-124-3p*, the other four top miRNAs were mapped in these three networks, respectively. The PPAR signalling gene-miRNA network consisted of 33 nodes and 43 edges (Figure 7D). In addition to *hsa-mir-26b-5p* and *hsa-mir-355-5p*, the *hsa-mir-124-3p* was mapped in the network and found interacts with the gene ACSL5 and ACADL. The *has-mir-29b-3p*, *hsa-mir-26b-5p* and *hsa-mir-355-5p* were the main miRNA regulators in the PI3K-Akt signalling (147 nodes, 146 edges), which interact with one of the two hub genes CCND2 (Figure 7E).

**TABLE 5:** List of genes identified for the selected KEGG pathways from Table 4, based on the upregulated DEGs in DMD-COs.

Hypertrophic cardiomyopathy (HCM) or Dilated cardiomyopathy				Arrhythmogenic right ventricular cardiomyopathy (ARVC)				PPAR signaling pathway			
Gene	Degree	Betweenness	Log_2_FC	Gene	Degree	Betweenness	Log_2_FC	Gene	Degree	Betweenness	Log_2_FC
CACNA1S	1	0	7.529686	CACNA1S	1	0	7.529686	FABP4	1	0	8.183692
ITGB3	12	384.8124	5.216347	ITGB3	5	324	5.216347	OLR1	4	368	3.924547
ACTC1	8	275.1753	4.075546	ACTN2	1	0	3.481939	ACSL5	2	120	3.631048
TNNC1	2	10.81398	3.798423	ITGA11	1	0	3.205193	CYP4A22	2	64	3.606022
MYBPC3	1	0	3.683695	ITGA8	1	0	2.541879	FABP1	1	0	3.506562
IGF1	11	464.726	3.348552	ITGA4	1	0	2.491913	APOA1	1	0	3.163481
ITGA11	5	74.9404	3.205193	ITGA10	1	0	2.380275	APOC3	4	336	3.091494
TNNT2	1	0	3.161717	RYR2	1	0	2.308044	APOA5	1	0	3.050644
TTN	1	0	3.012593	ITGA1	3	128	2.196014	PPARG	2	64	2.714461
ITGA8	2	6.105037	2.541879	ITGA9	1	0	1.860031	SLC27A6	1	0	2.327855
ITGA4	1	0	2.491913	LAMA2	1	0	1.754227	CYP4A11	1	0	2.147203
ITGA10	2	14.18264	2.380275	PKP2	1	0	1.751738	FABP3	1	0	2.004963
AGT	1	0	2.374724	SGCD	1	0	1.70417	PCK1	2	64	1.755388
RYR2	3	36.24091	2.308044	CACNA1C	3	128	1.674238	LPL	2	64	1.707849
TPM2	3	55.23974	2.265698	ITGA3	7	468	1.418763	ACOX2	1	0	1.541918
ITGA1	3	67.24618	2.196014	CACNB2	1	0	1.414398	PLTP	1	0	1.150143
TGFB3	1	0	2.166437	LMNA	1	0	1.408758	ACADL	3	264	1.10315
TPM1	4	36.31417	1.924547	ITGA5	3	128	1.083372	CD36	3	176	1.091012
ITGA9	3	28.60409	1.860031	CTNNA3	1	0	1.0382	EHHADH	1	0	1.0699
LAMA2	1	0	1.754227								
SGCD	6	151.5214	1.70417								
CACNA1C	5	70.16433	1.674238								
ITGA3	12	516.33	1.418763								
CACNB2	5	117.135	1.414398								
LMNA	6	146.4568	1.408758								
ITGA5	8	281.9914	1.083372								
											
**PI3K-Akt signaling pathway**								**ECM-receptor interaction**			
**Gene**	**Degree**	**Betweenness**	**Log_2_FC**	**Gene**	**Degree**	**Betweenness**	**Log_2_FC**	**Gene**	**Degree**	**Betweenness**	**Log_2_FC**
IBSP	1	0	9.292749	FGF1	1	0	2.120551	IBSP	1	0	9.292749
G6PC	1	0	5.863185	ANGPT2	1	0	2.080366	ITGB3	4	484	5.216347
GNG10	1	0	5.678754	PIK3CG	4	852	2.014266	COL1A1	1	0	3.760557
EFNA2	1	0	5.288848	ITGA9	1	0	1.860031	COL1A2	1	0	3.549527
ITGB3	2	1104	5.216347	TEK	1	0	1.858137	COL6A5	1	0	3.478834
EREG	1	0	4.161637	IL4R	1	0	1.853008	COL4A4	1	0	3.395479
FGF7	1	0	3.776064	NTF3	1	0	1.844582	VTN	1	0	3.254605
COL1A1	2	288	3.760557	OSMR	3	572	1.764551	ITGA11	1	0	3.205193
COL1A2	1	0	3.549527	PCK1	2	288	1.755388	COL6A3	1	0	2.866777
COL6A5	1	0	3.478834	LAMA2	1	0	1.754227	LAMA4	1	0	2.81102
HGF	2	288	3.423781	COL6A6	1	0	1.735629	THBS2	8	1375	2.746301
COL4A4	2	568	3.395479	THBS4	1	0	1.727874	THBS1	3	252	2.743205
IGF1	3	848	3.348552	COL4A1	3	572	1.700584	COL2A1	1	0	2.638159
VTN	1	0	3.254605	AREG	1	0	1.686065	ITGA8	2	128	2.541879
ITGA11	1	0	3.205193	LAMA3	1	0	1.655755	ITGA4	1	0	2.491913
FLT1	2	288	3.113672	ERBB4	1	0	1.624097	COL6A2	1	0	2.432485
TLR2	1	0	3.062241	FGFR4	1	0	1.533105	LAMB1	1	0	2.42084
IGF2	1	0	2.881069	FGF5	1	0	1.51941	COL9A1	1	0	2.39997
COL6A3	1	0	2.866777	PDGFD	1	0	1.425317	COL4A3	1	0	2.38678
CHRM2	2	568	2.85395	FGFR2	1	0	1.422263	ITGA10	1	0	2.380275
LAMA4	1	0	2.81102	ITGA3	1	0	1.418763	FN1	1	0	2.35285
THBS2	3	572	2.746301	PDGFRB	1	0	1.387124	COL9A3	1	0	2.309894
THBS1	2	1104	2.743205	JAK3	1	0	1.367528	ITGA1	2	648	2.196014
PDGFRα	1	0	2.643103	PDGFB	1	0	1.339525	ITGA9	1	0	1.860031
COL2A1	1	0	2.638159	CREB3L1	1	0	1.324319	LAMA2	1	0	1.754227
IL6R	4	852	2.555862	TNC	1	0	1.307584	COL6A6	1	0	1.735629
ITGA8	1	0	2.541879	PIK3AP1	1	0	1.297542	COL4A1	5	596	1.700584
ITGA4	1	0	2.491913	PDGFA	2	288	1.273971	LAMA3	1	0	1.655755
COL6A2	1	0	2.432485	VEGFC	1	0	1.252076	ITGA3	9	1411	1.418763
LAMB1	1	0	2.42084	GNB4	4	852	1.211026	TNC	1	0	1.307584
COL9A1	1	0	2.39997	KITLG	2	840	1.112549	CD44	1	0	1.254704
COL4A3	1	0	2.38678	COL4A2	1	0	1.112449	HSPG2	3	1209	1.243494
ITGA10	1	0	2.380275	ITGA5	1	0	1.083372	SV2B	1	0	1.221935
FN1	1	0	2.35285	CDKN1A	22	8484	1.073866	COL4A2	2	128	1.112449
COL9A3	1	0	2.309894	LPAR3	1	0	1.070672	CD36	1	0	1.091012
FGF23	2	288	2.284442	CCND2	23	6852	1.058491	ITGA5	2	128	1.083372
ITGA1	1	0	2.196014	ITGA10	1	0	2.380275				
NOS3	1	0	2.155993	ITGA8	1	0	2.541879				
FGF10	1	0	2.154001	CREB3L1	1	0	1.324319				

## Discussion

There is currently no cure for DMD patients. They are solely treated symptomatically *via* palliative therapies in combination with cardio-respiratory supporting devices in case of cardio-pulmonary complications–a major lethal cause in DMD patients. As DMD-related cardiomyopathy often manifested as hypertrophic or dilated heart due to cardiomyocyte deterioration followed by fibrosis and adipose tissue formation, novel therapeutic modality should be developed to prevent these pathological events from taking place in the heart. For this, gaining in-depth understanding on the human disease mechanisms is necessary. Unfortunately, limited accessibility to patient biopsy/autopsy and the inferiority of *in vitro* 2D cellular and animal models in fully recapitulating the human disease phenotype have precluded this scientific endeavour. Therefore, it is imperative to develop *in vitro* 3D human cardiac-mimics of DMD-relevance to bridge this scientific gap. The advent of hiPSC technology represents a paramount breakthrough for patient-specific model generation that can better mimic the individual phenotype. By using the isogenic-corrected controls (instead of healthy wild-type controls), we could compare the results at minimal genetic background variability.

In this study, we generated DMD-COs that functionally and transcriptionally modelled the cardiac evolution in DMD patients. Specifically, our model displayed a lack in proliferative capacity, cell death and a progressive deterioration of cardiomyocytes in early culture stages, followed by adipose tissue and fibrous-like tissue formation at later culture stage. We also observed a defect in physiological RyR-driven Ca^2+^ signals in DMD-CMs compared to isogenic-corrected and healthy controls. This further underpins the validity of our model since RyR dysfunction has also been implicated in dystrophic skeletal muscle cells (Andersson et al., 2012). In this work, dystrophic skeletal muscle was linked with leaky (skeletal muscle-type) RyR1 channels due to its oxidation. Hence, our work suggests that also the functional properties of (cardiac muscle-type) RyR2 channels may be affected in DMD patients, thereby contributing to cardiac pathophysiology. Probably as a consequence of the impaired Ca^2+^ handling and the dystrophic phenotype, DMD-COs showed a premature loss of the contractile properties compared to the controls independently from the CM:non-CM ratio, which is comparable in the two samples, as showed in the FACS analysis. These data also suggest the presence of a cardiomyocyte population within COs very close to the one physiologically contained in cardiac tissues (Lim, 2020).

Furthermore, DMD-COs displayed stable αACTN localization while α-, β-, γ- and δ-sarcoglycans became minimal present from day 14. In fact, the formed sarcoglycan complex possibly deteriorated within the DMD-COs over time due to its intrinsic DMD pathological phenotype. Although EHT models have been employed for investigating the DMD-CM contractile capability in the 3D context, the lack of several DAPC protein components, even in the healthy EHTs, hampers their usage as reliable genetic disease models (Gilbert et al., 2021). The fact that healthy organoids showed the presence of late cardiac differentiation markers as α-, β-, δ- and γ-sarcoglycans makes possible to model genetic cardiac diseases where sarcoglycans are missing. Furthermore, the cardiomyopathy phenotype development depends not only on the absence of dystrophin but also on the detrimental effects of non-cardiomyocyte subpopulations. Thus, 3D COs represent a superior *in vitro* model compared to EHTs in terms of cardiac maturation and environment.

These findings confirmed the formation of cardiac tissue within the organoids, which is also corroborated by the expression of atrial/ventricular markers and TNNT2 over time. Furthermore, the longer and sustained expression of the ventricular markers MYL2 and MYH7 suggests the ventricular-like identity of the CMs within these 3D models. The DMD-related phenotype is further confirmed by the detection of higher levels of NOX4 within the DMD-COs. Although NOX4 is constitutively active in healthy CMs at low levels inducing a cardioprotective effect, it has been demonstrated that high levels of NOX4 have severe detrimental effects. Indeed, the NOX4 accumulation contributes to the development of cardiovascular diseases by triggering an excessive ROS production. The endoplasmic reticulum stress marker GORASP2 increased over time in DMD-COs, while ARCN1 was more prominent in DMD-COs, but they were not co-localized with NKX2.5, suggesting other cell type than differentiating cardiomyocytes experienced high ER stress within the generated DMD-COs and DMD-Iso-COs. Moreover, we argued that the presence of GDF10 near the PDGFRα^+^ fibroblast/adipocyte progenitors could be a feedback regulation mechanism to inhibit pathological formation of adipose tissues in the DMD-COs, as GDF10 was not detected in DMD-Iso-COs where adipogenesis did not occur.

It is known by the literature that iPSC-derived CMs resemble foetal hearts from a metabolic standpoint and thus are qualitatively and quantitatively immature. After birth, the metabolism of CMs switches from glycolysis to a predominant use of oxidative phosphorylation to fulfil the energy demand of the contracting myocardium (Allen et al., 2016). In our model, the glycolytic marker PGK1 was strongly expressed in both DMD-COs and DMD-Iso-COs. We attempted to quantify the immunofluorescence signals for deleterious markers and correlated them with the aberrant fibrosis and adipogenesis observed only in DMD-COs. However, the results were affected by the heterogeneity in the cytoarchitectures, as the organoids were derived from hiPSC-EBs which might have undergone differential mesodermal induction. Still, comparing to pre-differentiated cardiomyocyte spheroids as well as engineered heart tissue (EHT) constructs, COs derived from hiPSC-EBs have the advantage of possibly containing other non-cardiogenic cells (as seen during heart development) that contribute to the adipogenesis and the fibrotic-like phenotype upon cardiomyocyte deterioration. Noteworthy that these pathological events were not observed in the DMD-Iso-COs controls. The reasons of the development of adipocytes and fibrotic-like tissues are still unclear and further experiments must be performed to elucidate the causes. We hypothesised that in the dystrophic environment the PDGFRα^+^ population, which includes fibroblast and adipose progenitors, could have a role in the onset of these detrimental events. In fact, the activation of the PDGFRα pathway in some adult cells must be sufficient to generate significant pro-fibrotic activity (Olson and Soriano, 2009). As reported in literature, dystrophic myocardium, due to the Ca^2+^ overload, is characterized by cell death and inflammatory response, which result not only in myocyte hypertrophy, atrophy/necrosis, fibrosis, but also in the replacement of heart muscle by connective tissue and fat (Flanigan, 2014). Intriguingly, contractile genes were upregulated in DMD-COs after 93 days of culture. The causes contributing to this upregulation in the diseased COs are still unknown. However, this phenomenon can be attributed to a compensatory mechanism, and we believe that other reasons are concurring with these results. Indeed, it is generally accepted that an abnormal elevation of the intracellular Ca^2+^ concentration in the dystrophin-deficient cardiomyocytes is a major secondary event, which contributes to disease progression and alters the contractile protein turnovers (Mareedu et al., 2021). Another possible explanation could be the foetal reprogramming occurring in diseased myocardium. This adaptive process is based on the suppression of adult and reactivation of foetal gene profile as a result of cardiac disease promoting the reversion of the contractile machinery to a more compliant foetal one (van der Pol et al., 2020). As reported in literature TNNC2, TPM1 and MYL2 are markers expressed at early developmental stages during cardiogenesis (Sheikh et al., 2015; England et al., 2017; Tsedeke et al., 2021). Furthermore, Cetinkaya et al. states that the ACTN1 expression in adult myocytes implies the activation of fetal pathways in patients affected by dilated cardiomyopathy (Cetinkaya et al., 2020). Moreover, MYBPC3 is not only expressed in adult but also in embryonic and neonatal hearts throughout the development (Jiang et al., 2015). These data could suggest the possible activation in DMD-COs of foetal genes in order to promote the turnover of sarcomeric proteins and counteract the cardiomyocyte derangement.

Through RNA sequencing analysis, we demonstrated that the DMD-COs generated on day 56 were valuable 3D cellular models to gain insight into the disease mechanism of DMD-associated hypertrophic/dilated cardiomyopathy, as well as adipogenesis and fibrosis. We focused on mapping out the functionally enriched pathways based on the differentially upregulated genes in DMD-COs as compared to DMD-Iso-COs, as well as their main miRNA regulators. Among the top five hub genes identified in the protein-protein interaction network, only HNF4A (log_2_FC = 1.89, *p* < 2.92e^−5^), UBD (log_2_FC = 2.69, *p* < 7.37e^−5^) and EGR1 (log_2_FC = 1.47, *p* < 5.31e^−12^) were significantly and differentially upregulated in DMD-COs. Despite HNF4A could be linked to cardiac differentiation and heart development (Duelen et al., 2017), we could not find in literature the association of these three hub genes with the development of cardiomyopathy, adipogenesis and fibrosis. We turned into looking at the identified miRNA regulators. The *hsa-mir-335-5p* was reported as a regulator of cardiac differentiation by upregulating cardiac mesoderm and cardiac progenitor commitments, potentially mediated through the activation of WNT and TGFβ pathways (Kay et al., 2019). In contrast, the upregulation of *hsa-mir-335-5p* was seen in fibrotic lung model (Honeyman et al., 2013). Additionally, a study showed that the *hsa-mir-29a-3p* and *hsa-mir-29b-3p* levels in cardiac tissue from patients with congenital heart disease was significantly increased, and the injection of *miR-29b-3p* into zebrafish embryos induced higher mortality and developmental disorders including cardiac malformation and dysfunction, as well as inhibition of cardiomyocyte proliferation by targeting NOTCH2 (Yang et al., 2020). Interestingly, delivery of *miR-29a-3p* has a beneficial effect in myocardial injury (Ren et al., 2021) and cardiac hypertrophy (Xie et al., 2020). Similarly, the *hsa-mir-26a/b-5p* was highly expressed in cardiac hypertrophy (Tang et al., 2020) and promoted myocardial infarction-induced cell death (Jung et al., 2021), yet overexpression of *miR-26a/b* attenuated cardiac fibrosis (Tang et al., 2017; Wang et al., 2019) and alleviated cardiac hypertrophy and dysfunction (Shi et al., 2021). Lastly, the *hsa-mir-124-3p* was reported to promote cardiac fibroblast activation and proliferation (Zhu et al., 2021), and its inhibition protects against acute myocardial infarction by suppressing cardiomyocyte apoptosis (Hu et al., 2019). Based on the duality effects of these miRNAs, the potential of these miRNAs as therapeutic targets for DMD-related cardiomyopathy need to be assessed carefully. Furthermore, the identified PI3K/Akt signalling pathway enriched in DMD-COs is interesting, as accumulating evidences showed that it plays a role in regulating the occurrence, progression and pathological cardiac fibrosis (Qin et al., 2021) and hypertrophy (Aoyagi and Matsui, 2011). These findings are encouraging and prompting us to investigate in future the potential of these miRNAs as therapeutic targets to inhibit the aberrant functional enrichments in DMD-COs. In turn, this will enable us to further validate DMD-COs as reliable *in vitro* 3D human cardiac models for DMD-related disease modelling, drug discovery and regenerative medicine.

In conclusion, we developed 3D human cardiac-mimics from DMD cell lines, which holds great relevance as these models reproduce *in vitro*, even if partially, the DMD-related cardiomyopathy (i.e. cardiomyocytes stress and deterioration) and disease progression (i.e. adipogenesis and fibrosis) in long-term cultures. By studying the transcriptomic dysregulations in DMD-COs versus the isogenic controls *via* RNA sequencing and *in silico* analysis, we have identified five miRNAs that were significantly and differentially expressed in late DMD-COs which could be associated with the functionally enriched hypertrophy and dilated cardiomyopathy, fibrosis and adipogenesis signalling pathways. These are encouraging findings showing the potential of these human cardiac-mimics as novel *in vitro* 3D cellular models for studying DMD cardiomyopathy. In future studies, adding endothelial cells to the EBs to generate 3D vascularized human cardiac models would also be highly valuable to investigate the cardiomyocyte-endothelial interplays in relation to DMD pathogenesis.

## Data Availability

The datasets presented in this study can be found in online repositories. The names of the repository/repositories and accession number(s) can be found below: NCBI [accession: GSE194297].
