# Supplementary material for: Long-term culture of patient-derived cardiac organoids recapitulated Duchenne muscular dystrophy cardiomyopathy and disease progression
Source: Front Cell Dev Biol. 2022 Aug 11;10:878311. doi: 10.3389/fcell.2022.878311 (PMC9403515; doi:10.3389/fcell.2022.878311)
Supplement: Supplementary file 1 [file DataSheet1.docx]

Supplementary Materials


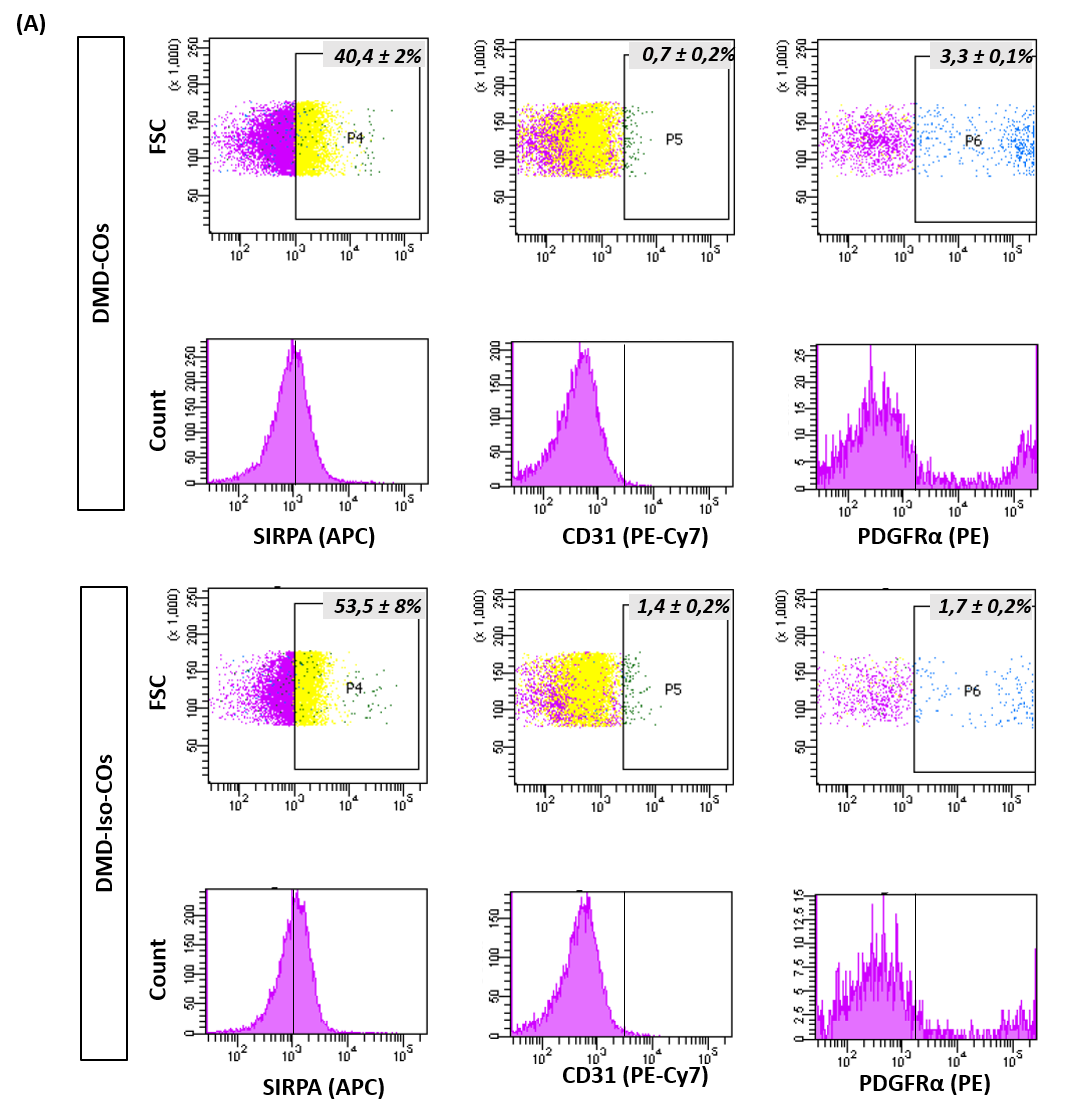


**Supplementary Figure 1.** **Flow cytometry analysis of DMD-COs and DMD-Iso-COs.** Representative flow cytometry analysis of **(A)** DMD-COs and **(B)** DMD-Iso-COs at day 14 of differentiation showing the percentage of SIRPA (cardiomyocytes), CD31 (endothelial cells) and PDGFRα (adipocytes/fibroblast precursors). Data are representative of three independent experiments (n = 3). Flow cytometry data are reported as mean ± SD.


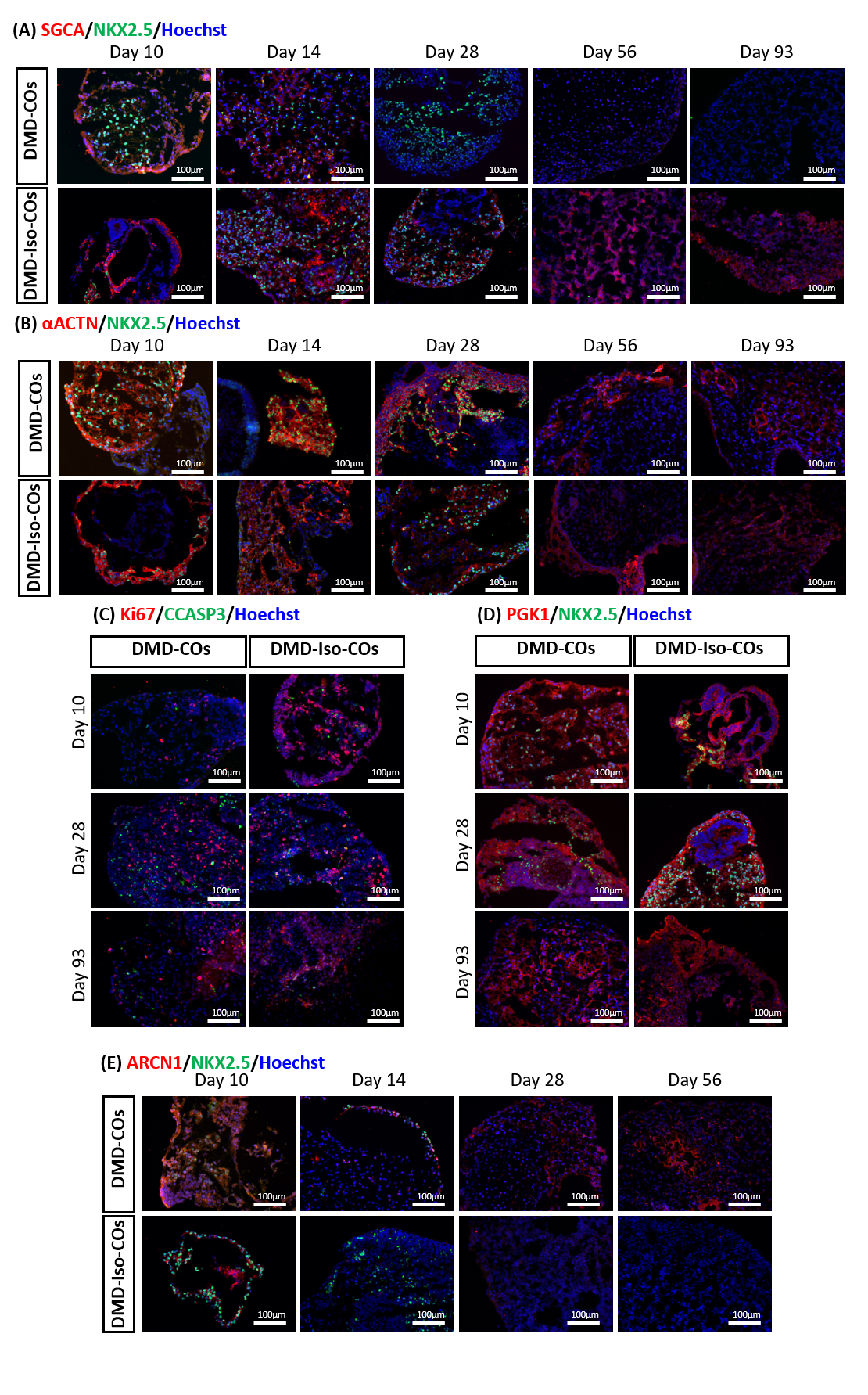


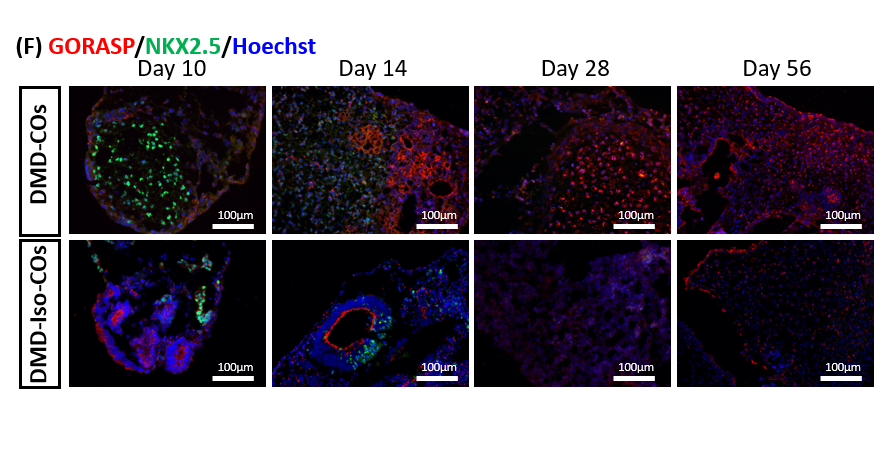


**Supplementary Figure 2. Additional immunofluorescence images of DMD-COs and DMD-Iso-COs**. Additional representative immunofluorescence images for: **(A)** α-sarcoglycan (SCGA)/NKX2.5, and **(B)** sarcomeric α-actinin (αACTN)/NKX2.5 on day 10, 14, 28 and 93**; (C)** Ki67/CCASP3 and **(D)** PGK1/NKX2.5 on day 10, 28 and 93**; (E)** ARCN1/NKX2.5 and **(F)** GORASP2/NKX2.5 on day 10, 28, 56 and 93 co-staining in DMD-COs and DMD-Iso-COs, respectively. Nuclei were counterstained with Hoechst. Data are representative of three independent experiments (n = 3); Magnification: 20X.


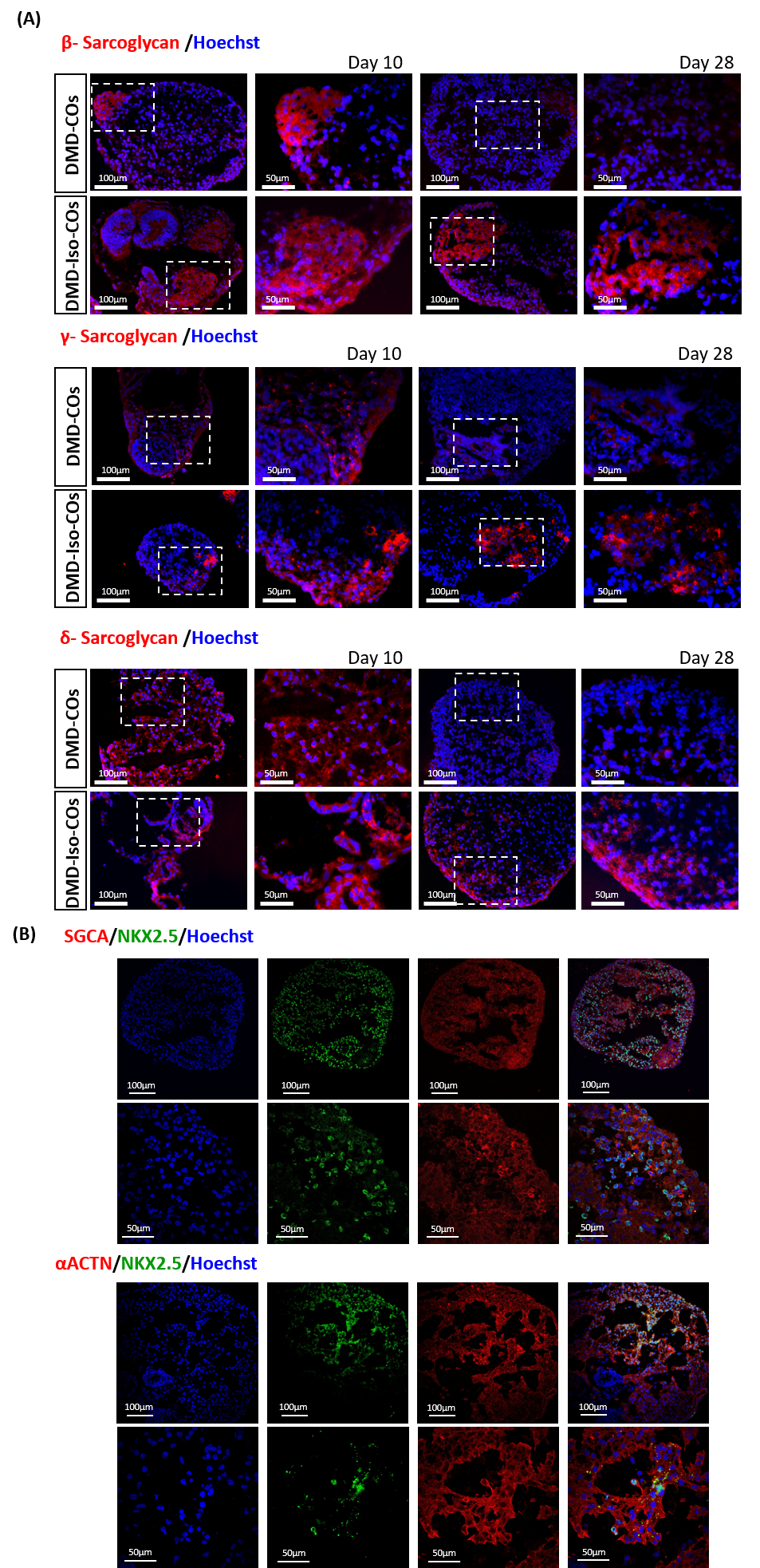


**Supplementary Figure 3. Sarcoglycans localization and sarcomeric pattern in DMD-COs and DMD-Iso-COs. (A)** Representative immunofluorescence images for β-, γ-, δ- sarcoglycan on day 10 and 28. Nuclei were counterstained with Hoechst. Data are representative of three independent experiments (n=3); Magnification: 20X and 40X. **(B)** Representative immunofluorescence images of the sarcomeric pattern in DMD-Iso-COs. Sections were stained for SGCA/NKX2.5 and αACTN/NKX2.5. Nuclei were counterstained with Hoechst. Data are representative of three independent experiments (n = 3); Magnification: 20X, 60X.


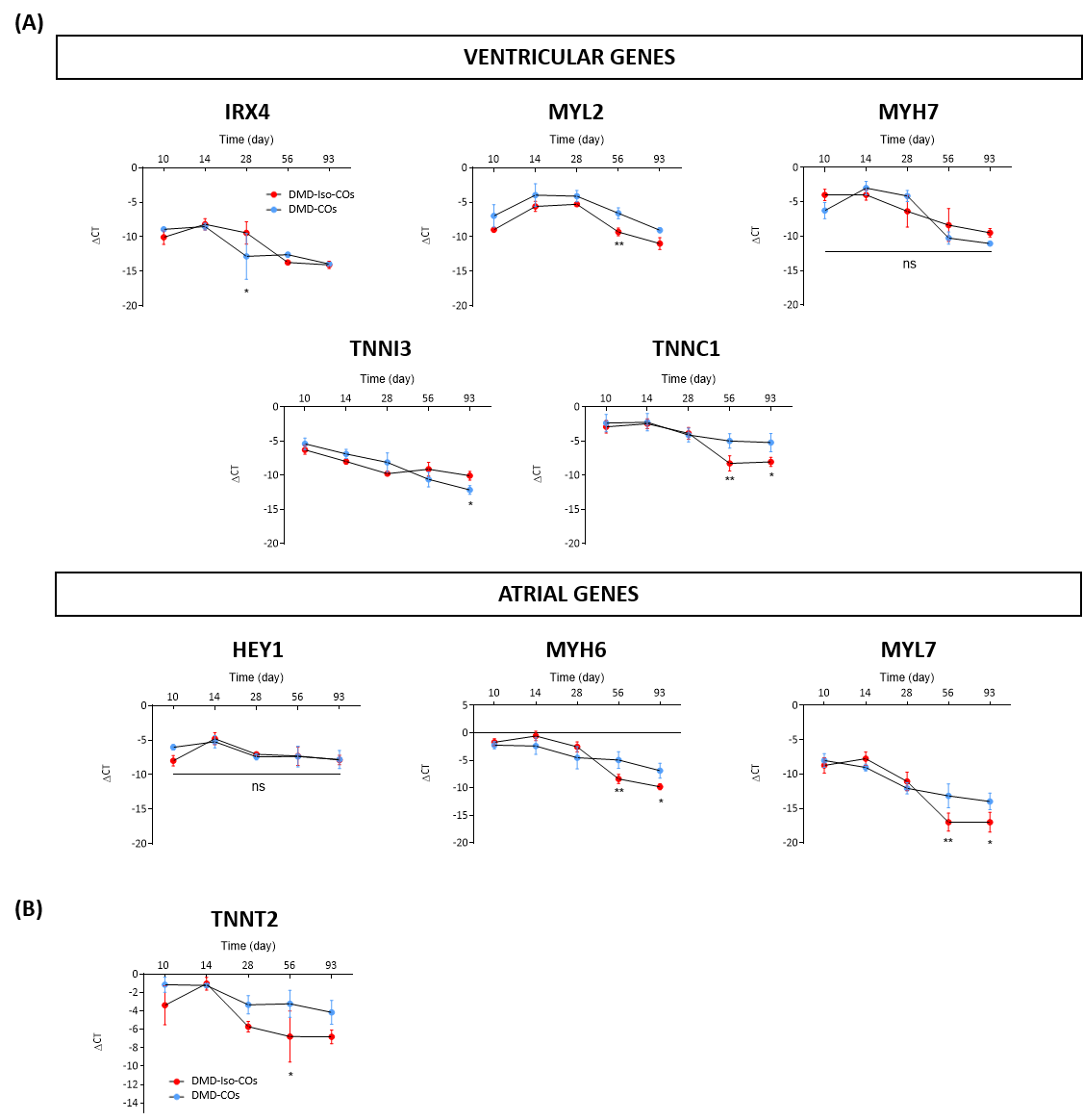


**Supplementary Figure 4. CM phenotype characterization of DMD-COs and DMD-Iso-COs over 93 days of dynamic culture. (A)** RT-qPCR analysis of representative ventricular and atrial gene markers expression in DMD-COs and DMD-Iso-COs at day 10, 14 28, 56 and 93. Data shown are mean + s.d. (n = 3, each pooled from ~10 organoids). Statistical analysis was performed by two-way ANOVA with Sidak’s multiple comparisons: *p<0.05, **p<0.01. **(B)** RT-qPCR analysis of TNNT2 gene expression in DMD-COs and DMD-Iso-COs at day 10, 14 28, 56 and 93. Data shown are mean + s.d. (n = 3, each pooled from ~10 organoids). Statistical analysis was performed by two-way ANOVA with Sidak’s multiple comparisons: *p<0.05.


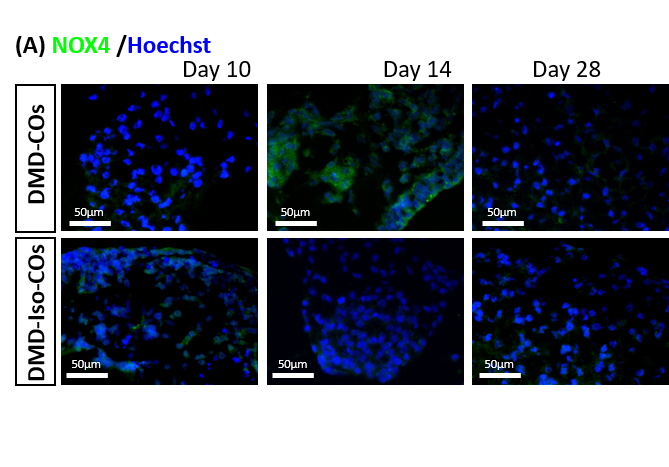


**Supplementary Figure 5. NOX4 localization in DMD-COs and DMD-Iso-COs.** **(A)** Representative immunofluorescence images for NOX4 on day 10, 14 and 28. Nuclei were counterstained with Hoechst. Data are representative of three independent experiments (n = 3); Magnification: 20X and 40X.


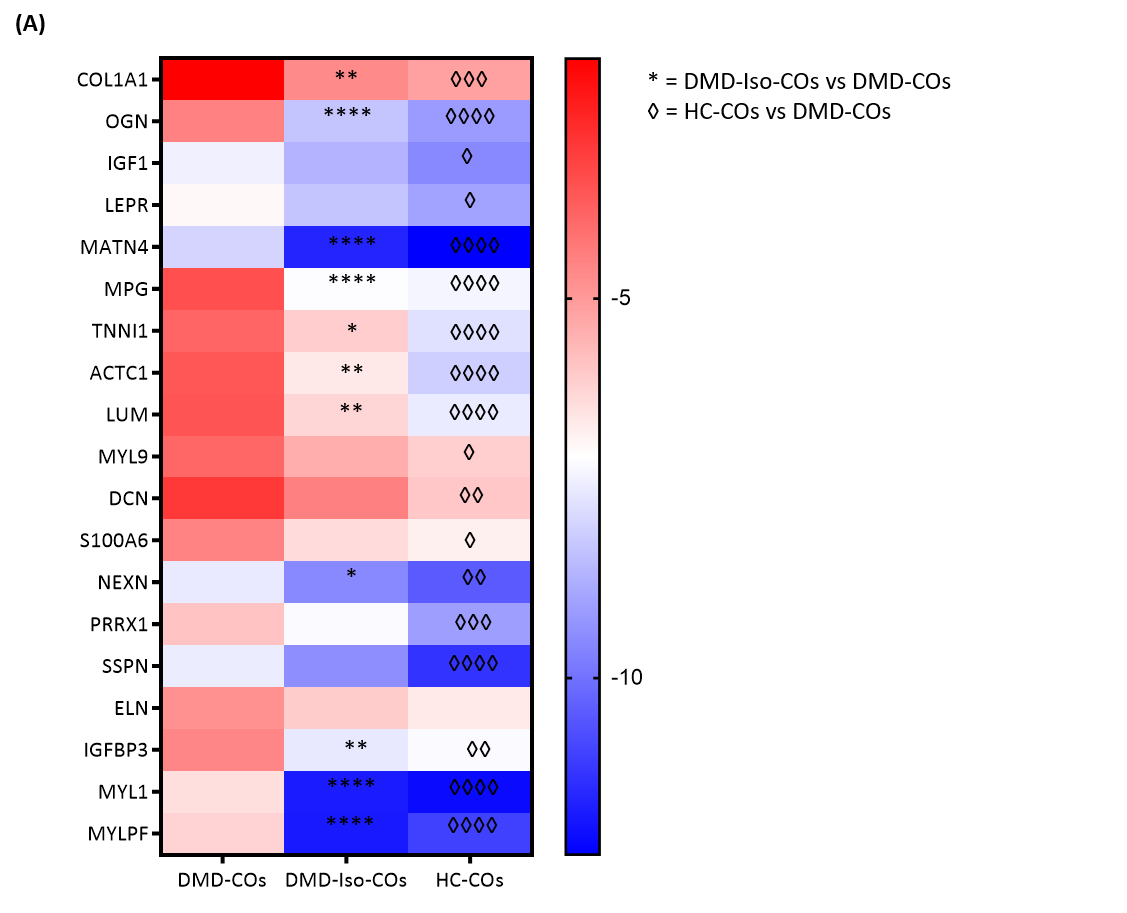


**Supplementary Figure 6. RNAseq-derived** **TOP30 DEGs validation by RT-qPCR on DMD-COs, DMD-Iso-COs and HC-COs. (A)** RT-qPCR analysis of representative TOP30 DEGs obtained from RNAseq analysis in DMD-COs, DMD-Iso-COs and HC-COs. Data shown are mean + s.d. (n = 3, each pooled from ~10 organoids). Statistical analysis was performed by two-way ANOVA with Tukey’s multiple comparisons: *p<0.05, **p<0.01, ***p<0.001, ****p<0.0001.
